# Supplementary material for: Advancing respiratory–cardiovascular physiology with the working heart–brainstem preparation over 25 years
Source: J Physiol. 2022 Apr 7;600(9):2049–75. doi: 10.1113/JP281953 (PMC9322470; doi:10.1113/JP281953)

Appendix to Paton JFR et al., Advancing respiratory-cardiovascular physiology with the working heart-brainstem preparation over 25 years, The Journal of Physiology.

Building a WHBP set-up can be difficult, because key elements are not readily available for purchase from scientific equipment distributors. With this appendix, we aim at facilitating this process, by providing a list of the key elements necessary, with suggested manufacturer and specifications for those that are commercially available, and with technical drawings and pictures for parts that need to be custom-made. This is not an exhaustive list, but the minimum required to build a WHBP set-up. Also, please be aware that these are merely suggestions, each set-up is unique. Investigators have to adjust their WHBP set-up to their experimental requirements, as seen from the large diversity of WHBP variations and experimental needs in the review for which this is an appendix.

All custom-made parts provided here were 3D designed and generated by Clément Menuet, INMED, Marseille, France, using the SOLIDWORKS 2017 SP1.0 software (Dassault Systèmes). Please feel free to contact Clément if you have any question, or if you want to obtain the original 3D files to modify them to generate new technical drawings that better fit your needs: clement.menuet@inserm.fr.
Also, the recordings aspects (electrodes, pressure transducer, electrophysiology amplifiers, hardware and software, optogenetic/chemogenetic tools etc.) are not developed here, but suggestions can be provided upon request.

Equipment to set-up the WHBP that is available commercially:

- **Peristaltic Pump:** most labs use Watson Marlow 502S, 505S or 520S peristaltic pumps. Then, different pump heads can be used, and use of a multichannel cassette (at least 3) is recommended to enable driving both inputs and outputs of the WHBP with one pump. The size and more importantly the number of rollers present in the pump head make a difference. The more rollers there are the better, as the flow generated is more laminar. Pump heads with 12 rollers are available and ideal for the Watson Marlow pumps, although they can be difficult to find. Pump heads with 8 rollers, which are more common, also work. When choosing a pump head, it is important to consider the flow rate indicated by the manufacturer. For the WHBP, typical flow rates are ~18-20 ml/min in adult mice, ~10 ml/min in P10 rats, ~15 ml/min in P21 rats, and ~20-25 ml/min in P30-P35 rats, so the pump head must be chosen accordingly. If needed, it is interesting to note that multiple tubes can be used on the pump head and connected into one for the input, to maximise the flow rate and its laminar aspect, and diminish the work done by the pump, for instance when using a small pump head with few rollers with a maximum flow rate close to the ones necessary for the WHBP.
- **Pump tubing:** purchasing specific tubing sold by the pump manufacturer is usually necessary, so that the tubing fits the pump head used. Similarly to tubing for input below, it is best to choose pump tubing in a material that is bio-compatible and with low permeability to gas. Marprene material is often available for pump tubing and recommended. Also, it is common practice to use a number of pump tubes for the output (preparation to aCSF flask) that is superior to the number of pump tubes used for the input (aCSF flask to preparation). This way, aCSF should never accumulate in the preparation chamber.
- **Tubing for input – aCSF flask to preparation:** favour tubing with low permeability to gas, which are bio-compatible and resistant, and thick enough to transmit flow rate efficiently. An example of input tubing that works is the Masterflex Tygon E-Lab E-3603 L/S 14 (tubing internal diameter 1.6 mm / 0.063 inches, hose barb 1/16 inches). Importantly, input tubing in the WHBP set-up must be reduced to the minimum length

possible, to minimise damping of flow rate / pressure provided by the pump. The less space for aCSF between the flask and the preparation, the better. This is especially important in older rats. Also, note that it is important to ground the input aCSF, as close to the preparation as possible. For this, it is for instance possible to put a small piece of stainless steel tube in the input tubing just before it reaches the double lumen catheter, with part of this stainless steel tube that is connected to the set-up Faraday cage *via* a dedicated wire cable.

- **Tubing for output – preparation to aCSF flask:** any bio-compatible tubing should work. An example of output tubing that works is the Masterflex Platinum-Cured Silicone Precision Tubing L/S 14 (tubing internal diameter 1.6 mm / 0.06 inches, hose barb 1/16 inches). Also, similarly to the input, note that it is important to ground the output aCSF, as close to the preparation as possible. For this, it is for instance possible to connect the two stainless steel tubes used as outputs of the recording chamber (see below) to the set-up Faraday cage *via* dedicated wire cables.
- **High pressure filter holder:** this part is necessary to hold the filter that prevents particles larger than 25 µm from entering the WHBP vasculature. An example that works is the Merck high pressure filter holder, 25 mm, stainless steel, model XX4502500. In this holder, you can use polypropylene filters, 25 mm diameter, 25 µm retention threshold, model PP2502500. To ensure no leakage, we recommend using a flat O-ring or Teflon at the junction of the two pieces of the high pressure filter.
- **Double lumen catheter:** this part is necessary to bring aCSF into the preparation vasculature (first lumen), and to record perfusion pressure, the "blood pressure" of the preparation (second lumen). Choosing the right lumen sizes is important to optimise the perfusion/pressure ratio. A model that works is the BARD PowerPICC polyurethane 5F double lumen catheter (each lumen is 18G), Ref 6275118. This catheter (which can be cut for shorter length) will most likely not fit directly into the descending aorta to be cannulated, so a cut pipette tip for instance can be used.

Equipment to set-up the WHBP that is not available commercially (or hard to find):

Please note that for all these custom-designed parts, we provide here the 2D technical drawings in the A0 format, which contain all dimensions (in mm) and information necessary for machining the parts. We recommend using polymethyl methacrylate (PMMA) to make these parts. It can be raw for the parts that don't require full transparency, like the recording chamber and the heat exchanger, or polished (usually at an extra cost) to reach full transparency for the bubble traps. We also provide a picture of the parts made with these exact technical drawings, to enable better visualisation of how these parts look like.

- **Heat exchanger:** this part will require machining the chamber, and fixing a stainless steel tube that crosses the chamber, as shown on the technical drawing below. The tube is best fixed to the chamber with a polyurethane glue. Then, a standard open bath heating immersion circulator can be connected to the heat exchanger input and output via tubing, to bring heated water around the stainless steel tube, to warm the aCSF flowing through the stainless steel tube.

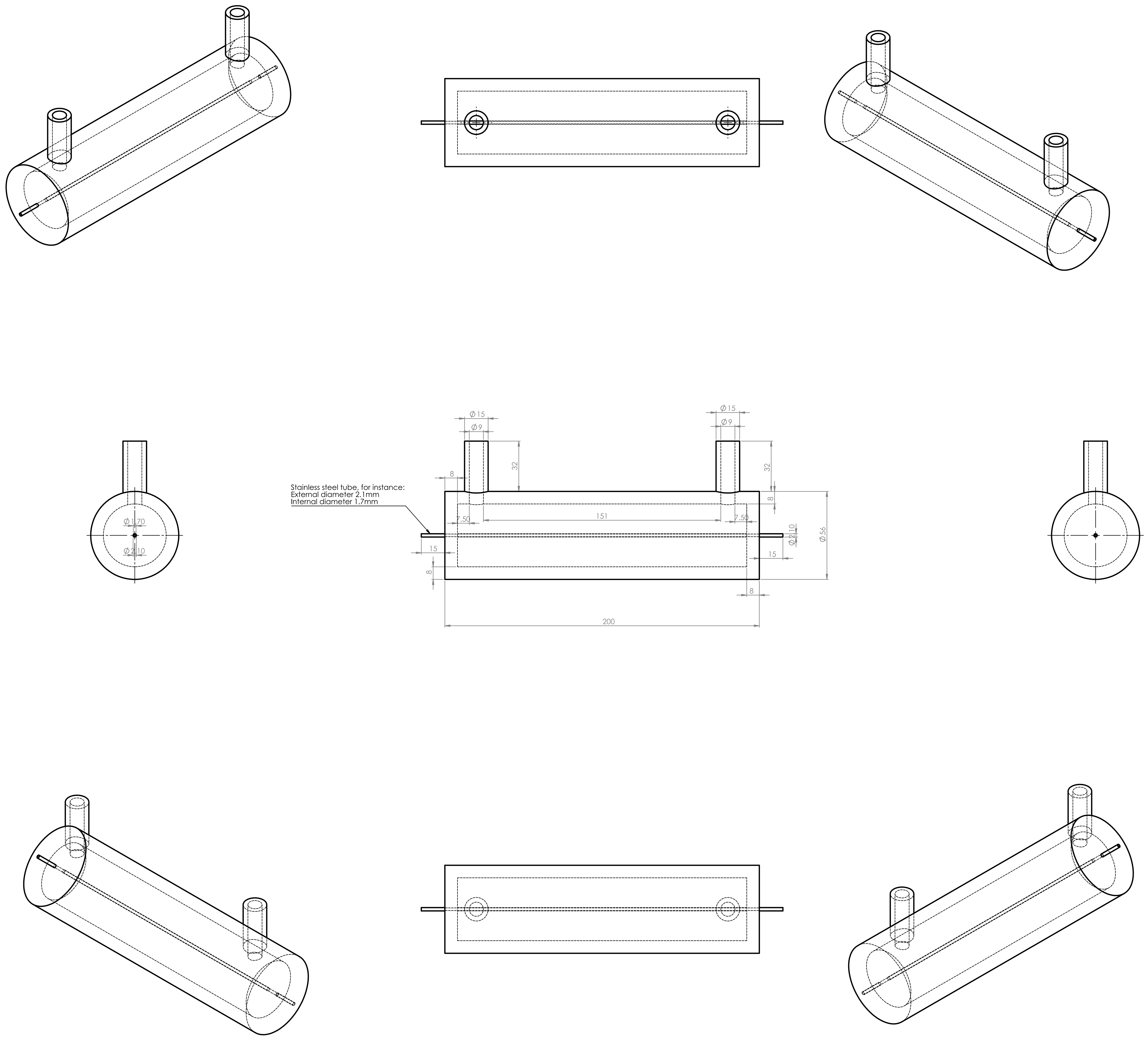

DIMENSIONS IN MILLIMETER	MATERIAL RECOMMENDED: PMMA FOR THE BODY PART, STAINLESS STEEL FOR THE INTERNAL TUBE

	NOM	SIGNATURE	DATE
AUTEUR			
VERIF.			
APPR.			
FAB.			
QUAL.			

TITRE: Heat exchanger 2D plan

No. DE PLAN — A0

SCALE: 1:1 — FEUILLE 1 SUR 1

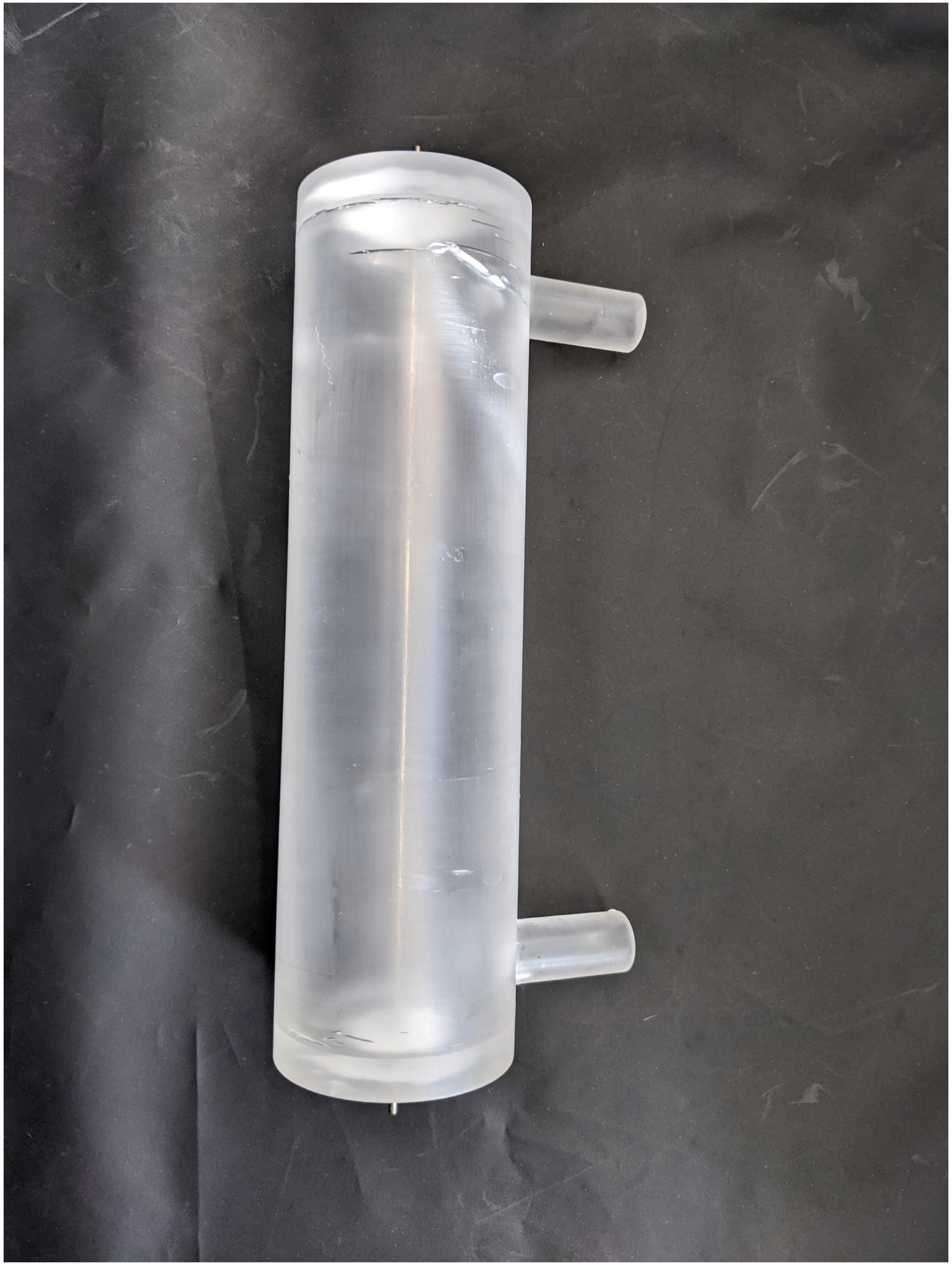

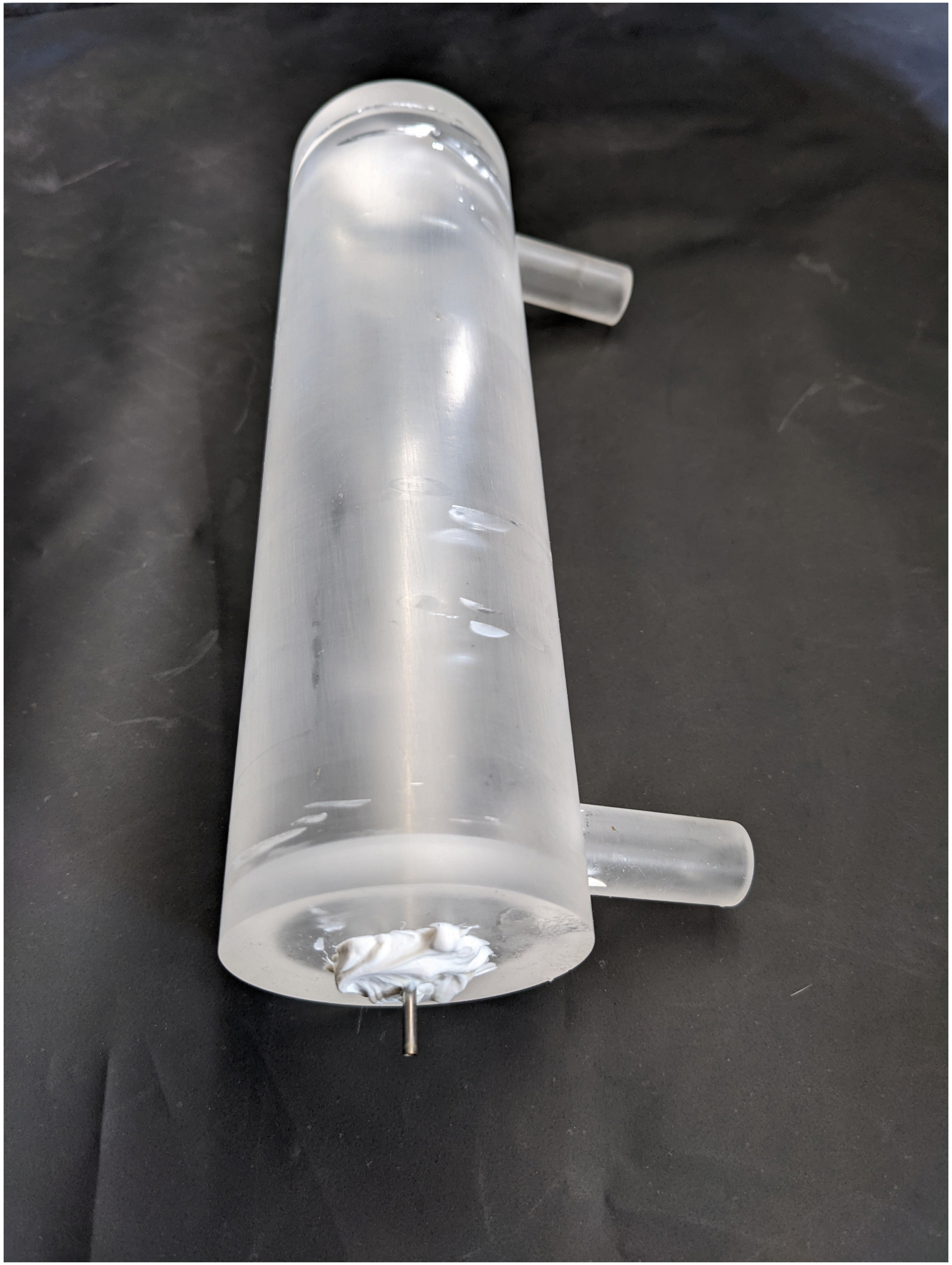

- **Bubble trap:** this part is made of two pieces, which are fixed together thanks to screws, with three dedicated threads on the bottom piece (M5 size, see technical drawing). We recommend using screws made in nylon. Also, both pieces contain threads (NPT 1/8", see technical drawings) that are designed to screw polypropylene connectors for the input and output tubing for the aCSF flow, and to a three-way tap to regulate the liquid level in the bubble trap (see picture below). The thread size was chosen to best fit tubing connectors of standard sizes (threaded to hose for tubing, threaded to luer for the three-way tap). We recommend using Teflon tape at the junction of all pieces, top and bottom pieces of the bubble trap, and threads with the connectors. For best results in manufacturing the threads, advice should be given to the machining manufacturer to first bore the threads with a drill, and then shape them with the appropriate tap.

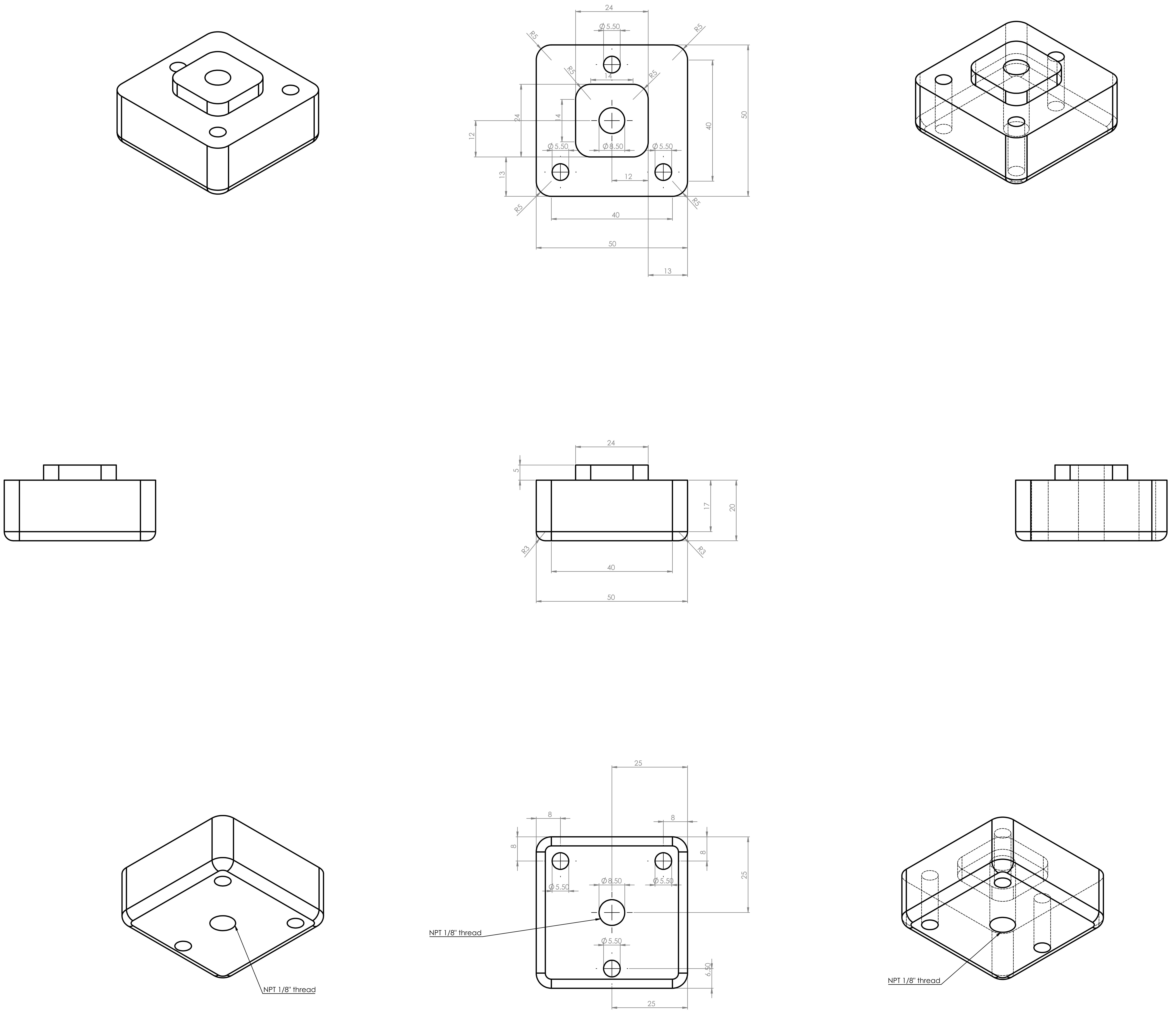

DIMENSIONS IN MILLIMETERS

RECOMMENDED MATERIAL: PMMA WITH POLISHED FINISHING FOR TRANSPARENCY

REVISION

	NOM	SIGNATURE	DATE
AUTEUR			
VERIF.			
APPR.			
FAB.			
QUAL.			

TITRE: Bubble trap top part 2D plan

No. DE PLAN

A0

SCALE: 5:1

FEUILLE 1 SUR 1

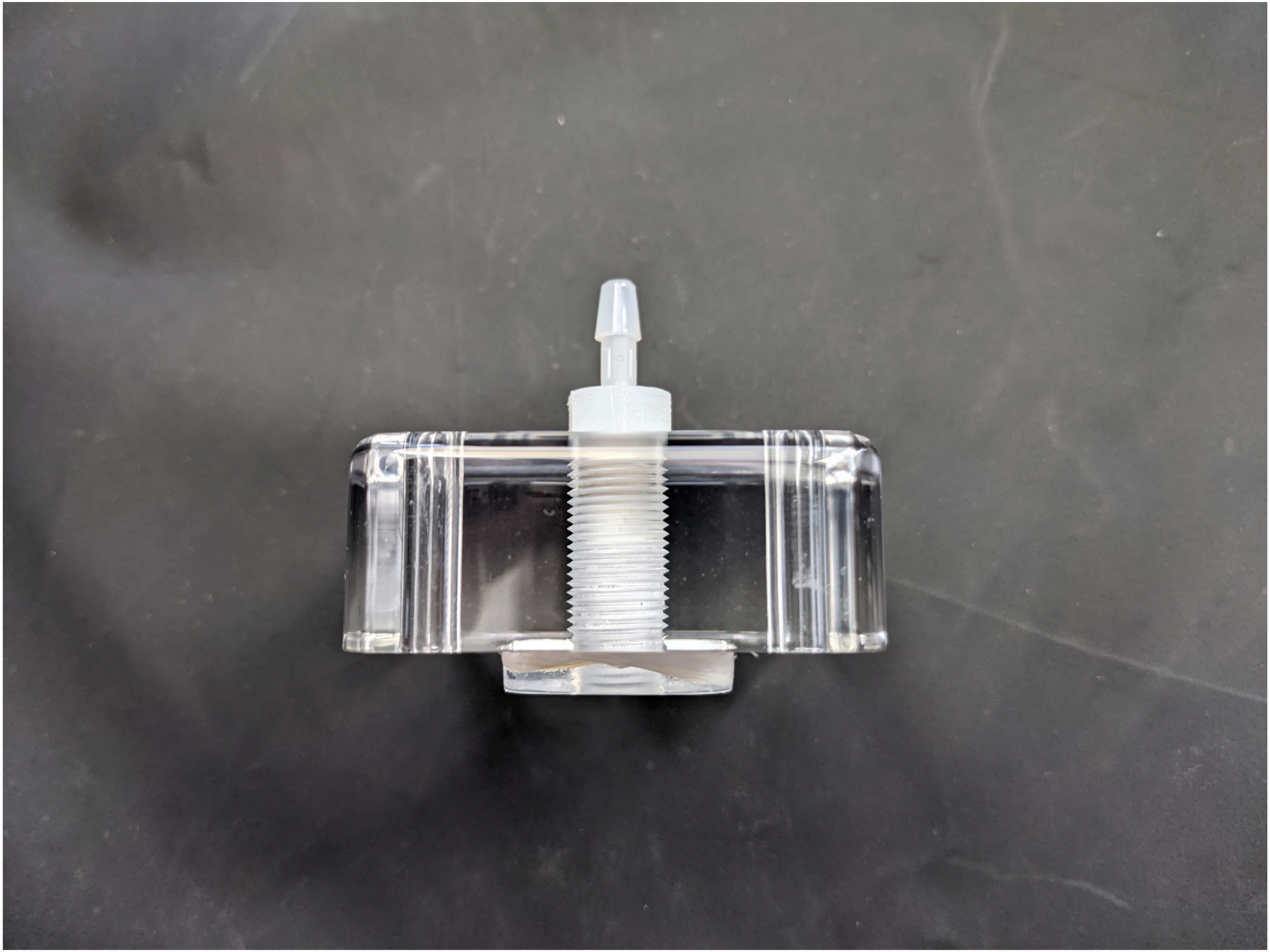

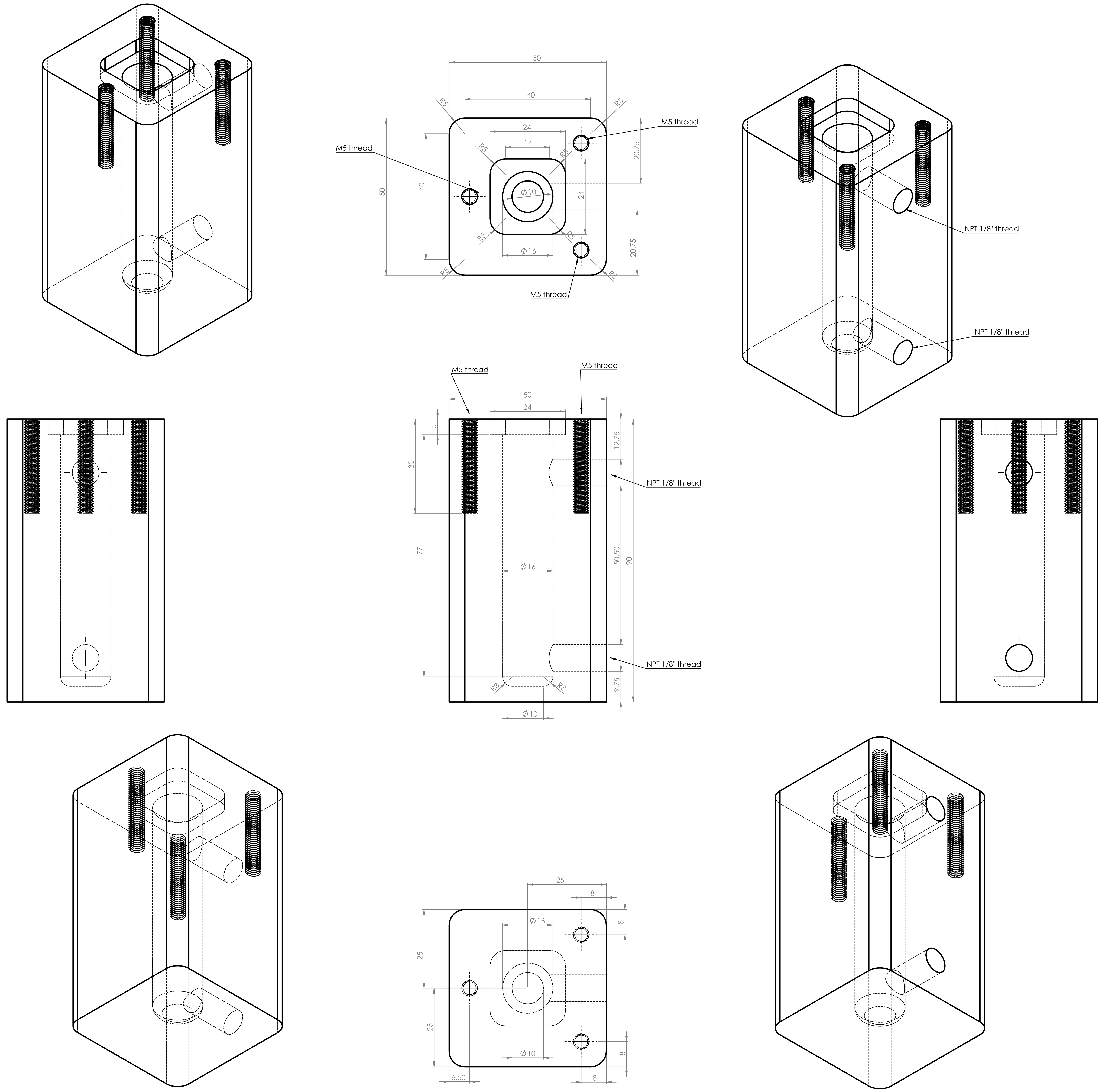

DIMENSIONS IN MILLIMETRES	RECOMMENDED MATERIAL: PMMA WITH POLISHED FINISHING FOR TRANSPARENCY					REVISION
	NOM	SIGNATURE	DATE			TITRE: Bubble trap bottom part 2D plan
AUTEUR						
VERIF.						
APPR.						
FAB.						
QUAL.						A0
						SCALE: 2:1
						FEUILLE 1 SUR 1

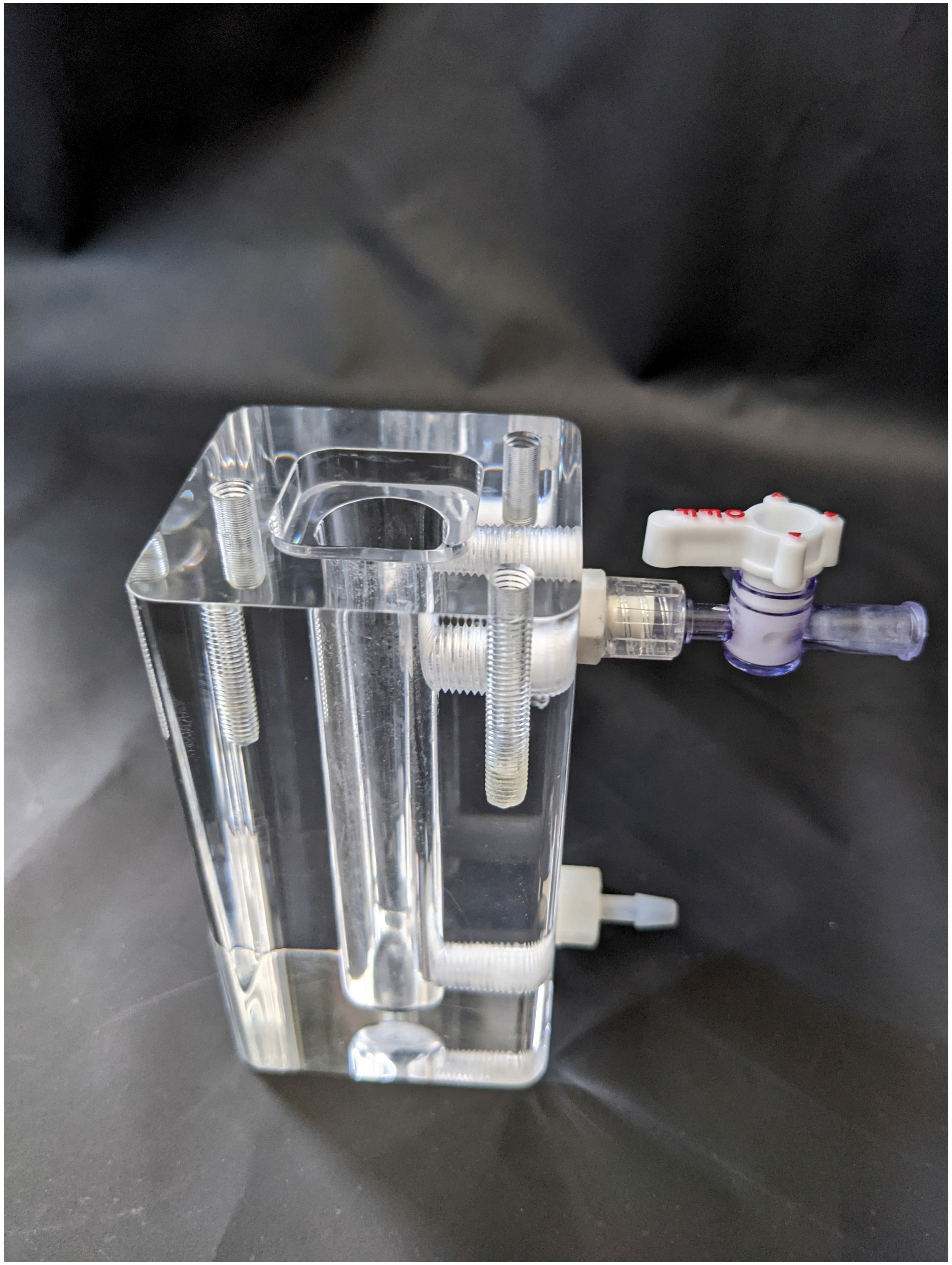

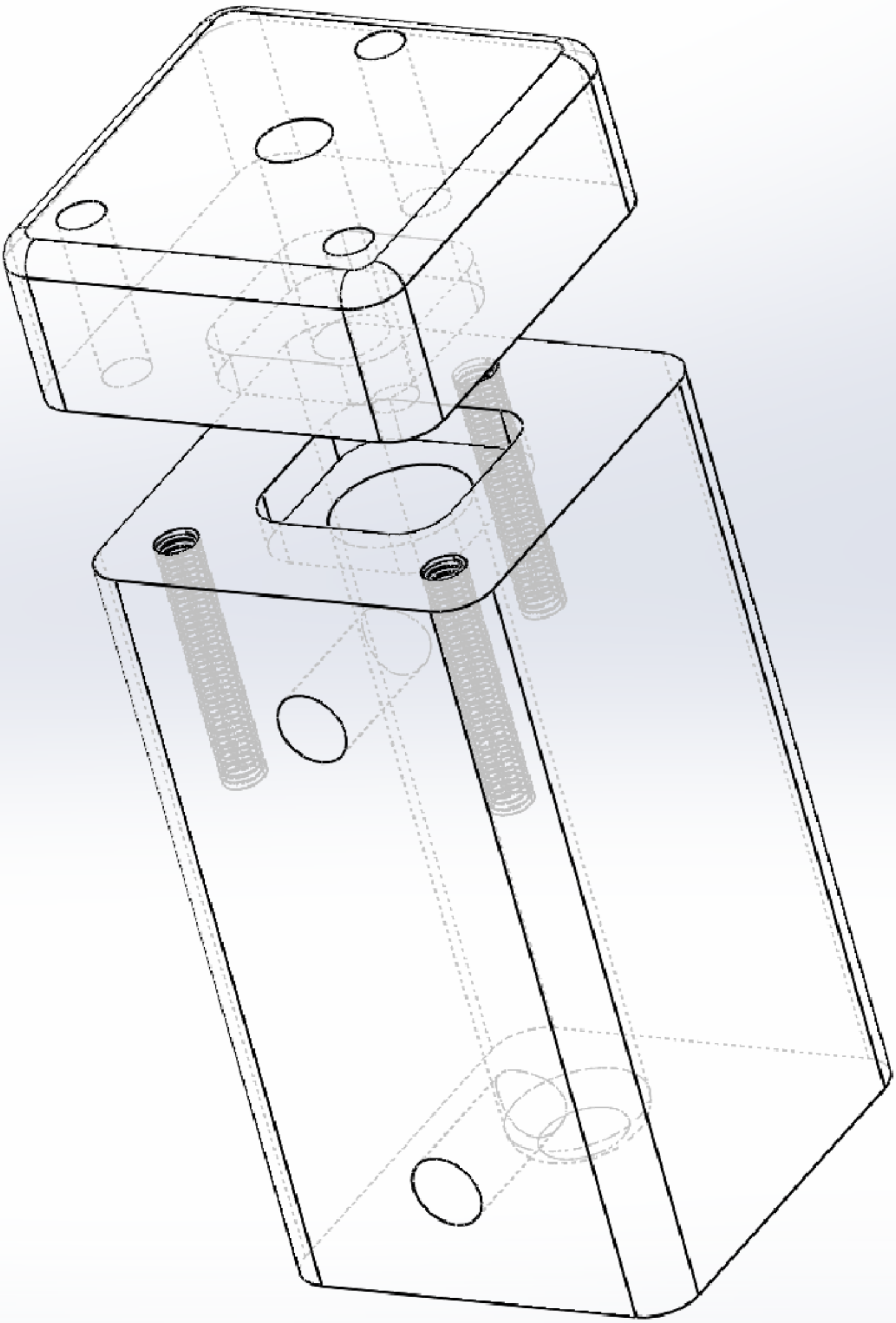

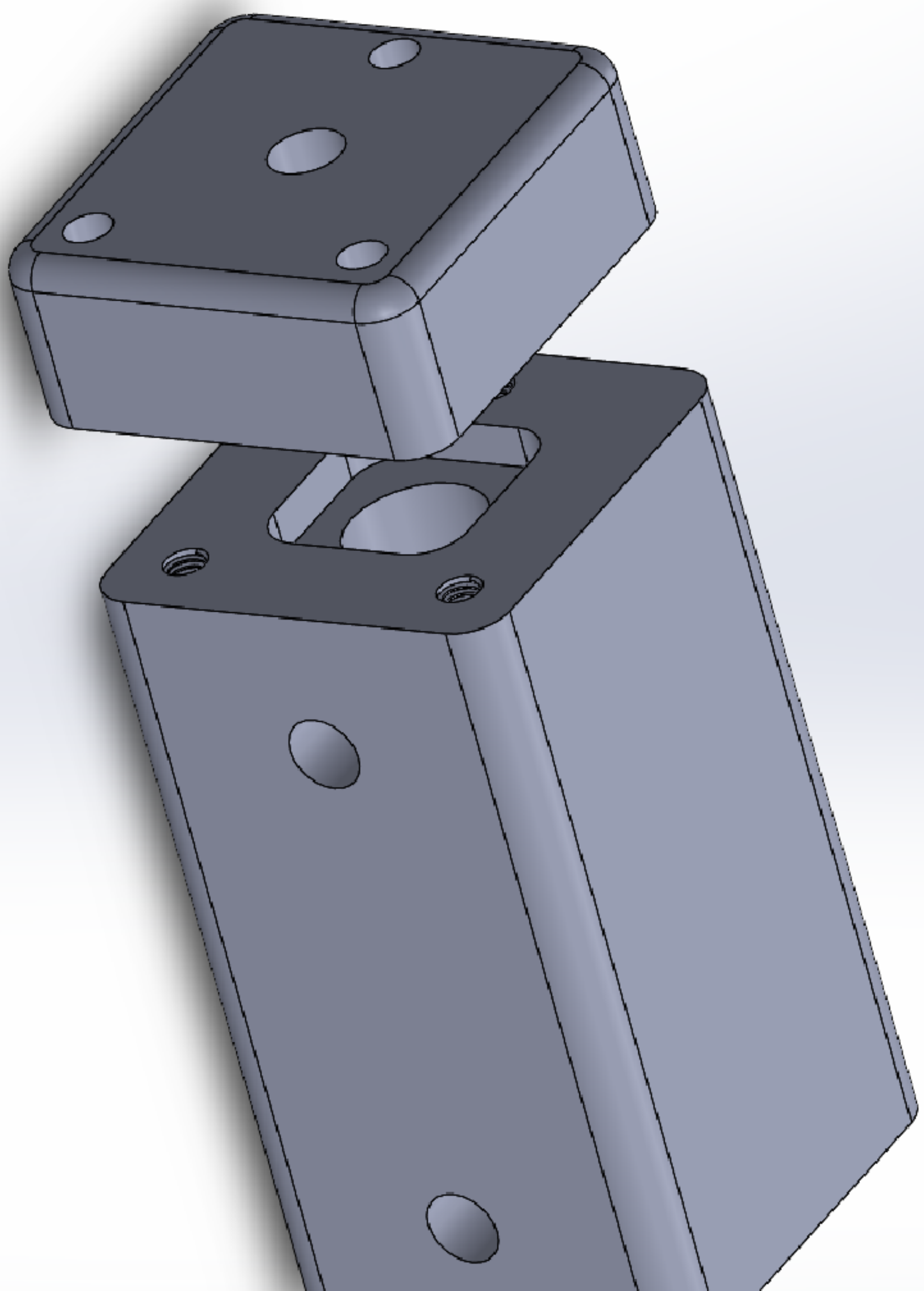

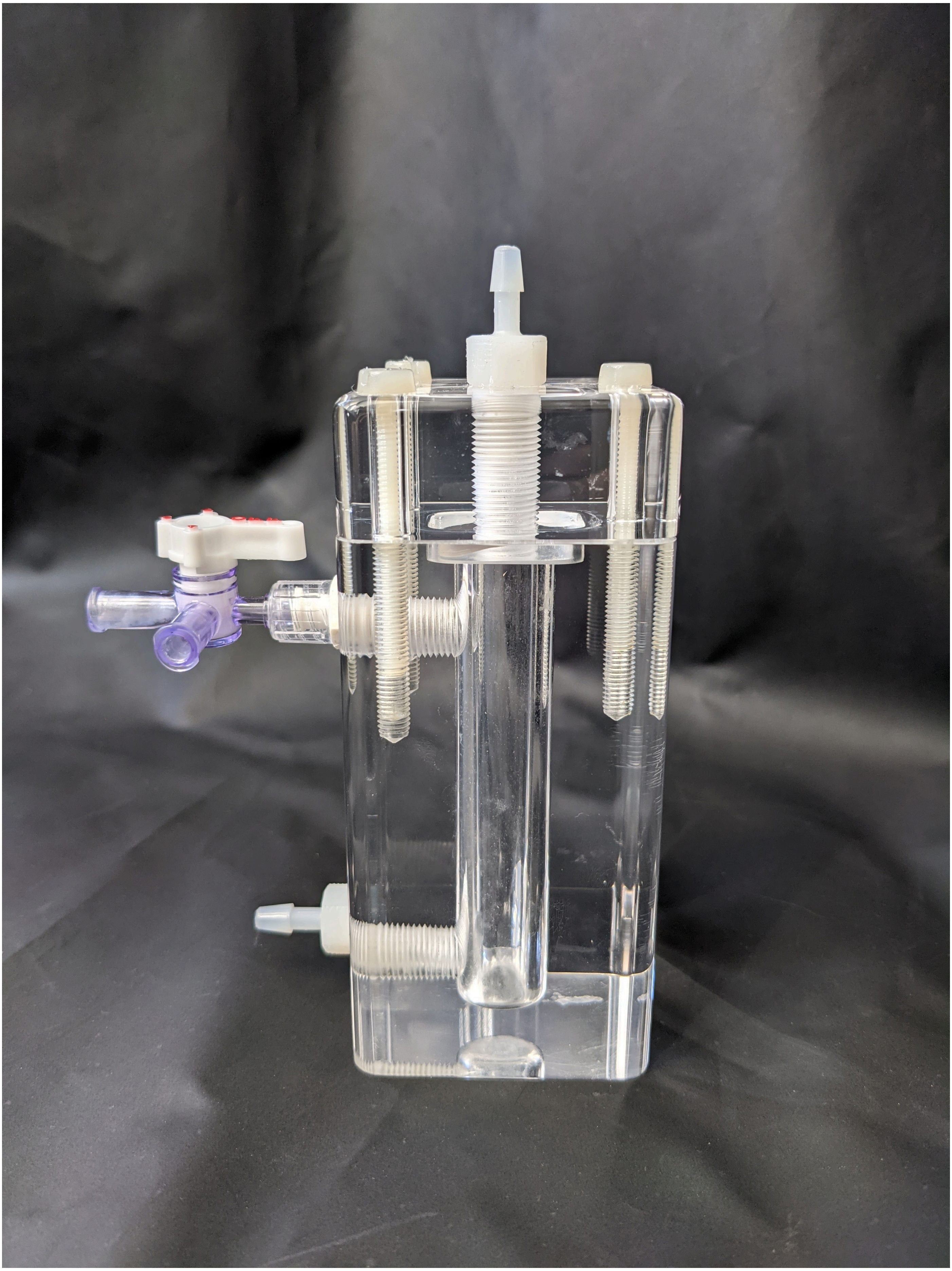

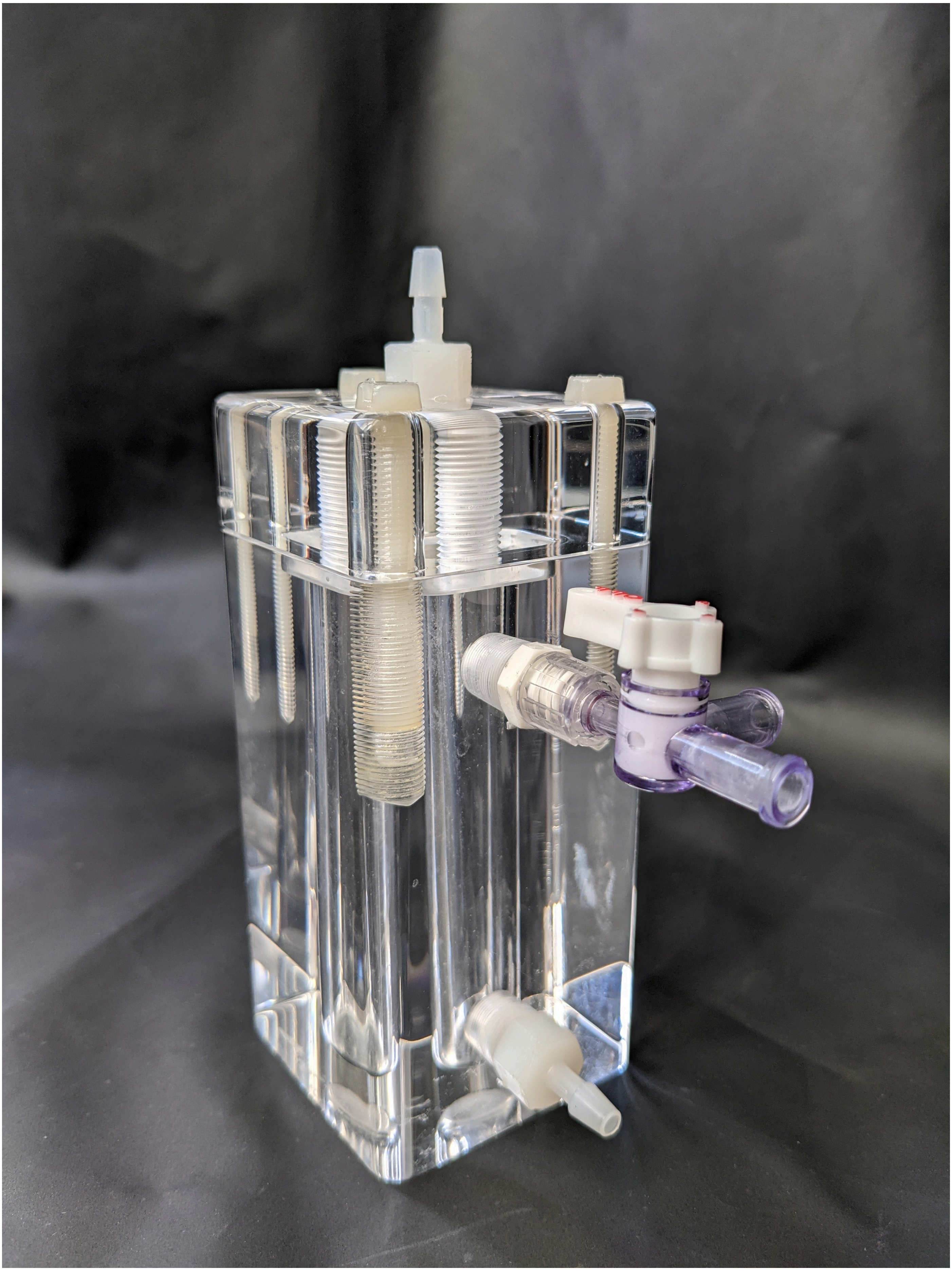

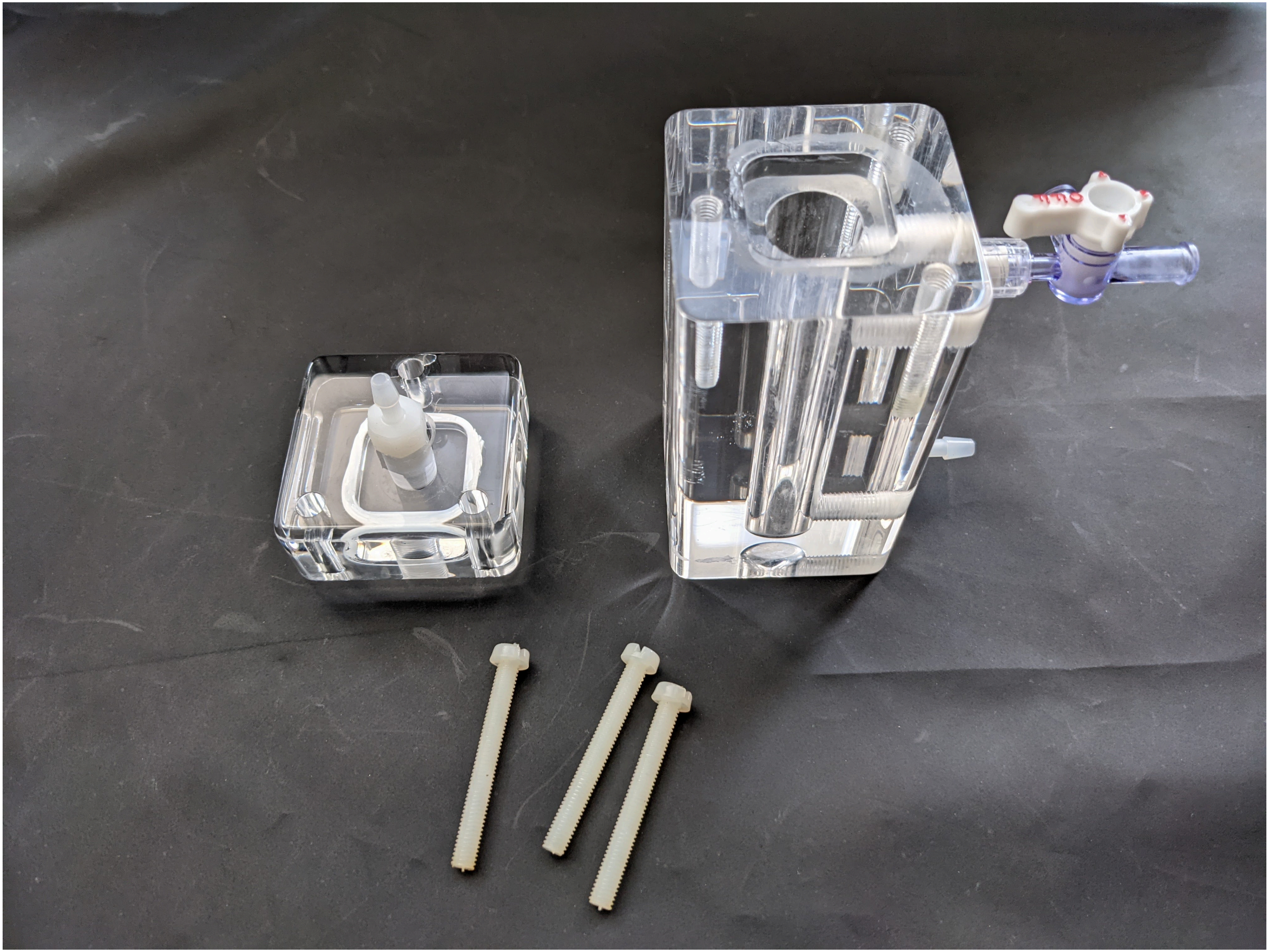

- **Recording chamber:** this part is made of 5 pieces, which assemble thanks to screws (see technical drawings below). We recommend using butterfly screws, for easiness of use, and made in nylon, so that they don't damage the material of the recording chamber pieces and don't deteriorate with the exposition to aCSF. Similarly to above, for best results in manufacturing the threads, advice should be given to the machining manufacturer to first bore the threads with a drill, and then shape them with the appropriate tap.
  The design of this recording chamber was made to enable fixing the head of the preparation in a similar way to a stereotaxic frame, with the possibility to modulate the height of the mouth bar, in particular to enable positioning the preparation for either the ventral or the dorsal approach of the brainstem. To hold the body of the preparation, we recommend building a platform in silicone (for instance using Sylgard), to enable pinning the body of the preparation in different positions and at the height desired for the experiments to be done. The preparation will therefore not touch the floor of the chamber, reducing electrical noise by creating a break in aCSF contact with the output. The ear bars were designed for juvenile rats, and should also work on adult mice. For the aCSF output, we recommend gluing (polyurethane glue) stainless steel tubes (which can be used for grounding the output aCSF, cf. above), and then using polypropylene connectors to connect to the output tubing. The recording chamber can be positioned on the set-up air table by taping small rubber feet under the chamber bottom plate, and can be fixed using magnets surrounding the chamber bottom plate. A schematic showing assembling of the pieces forming the recording chamber is provided below.

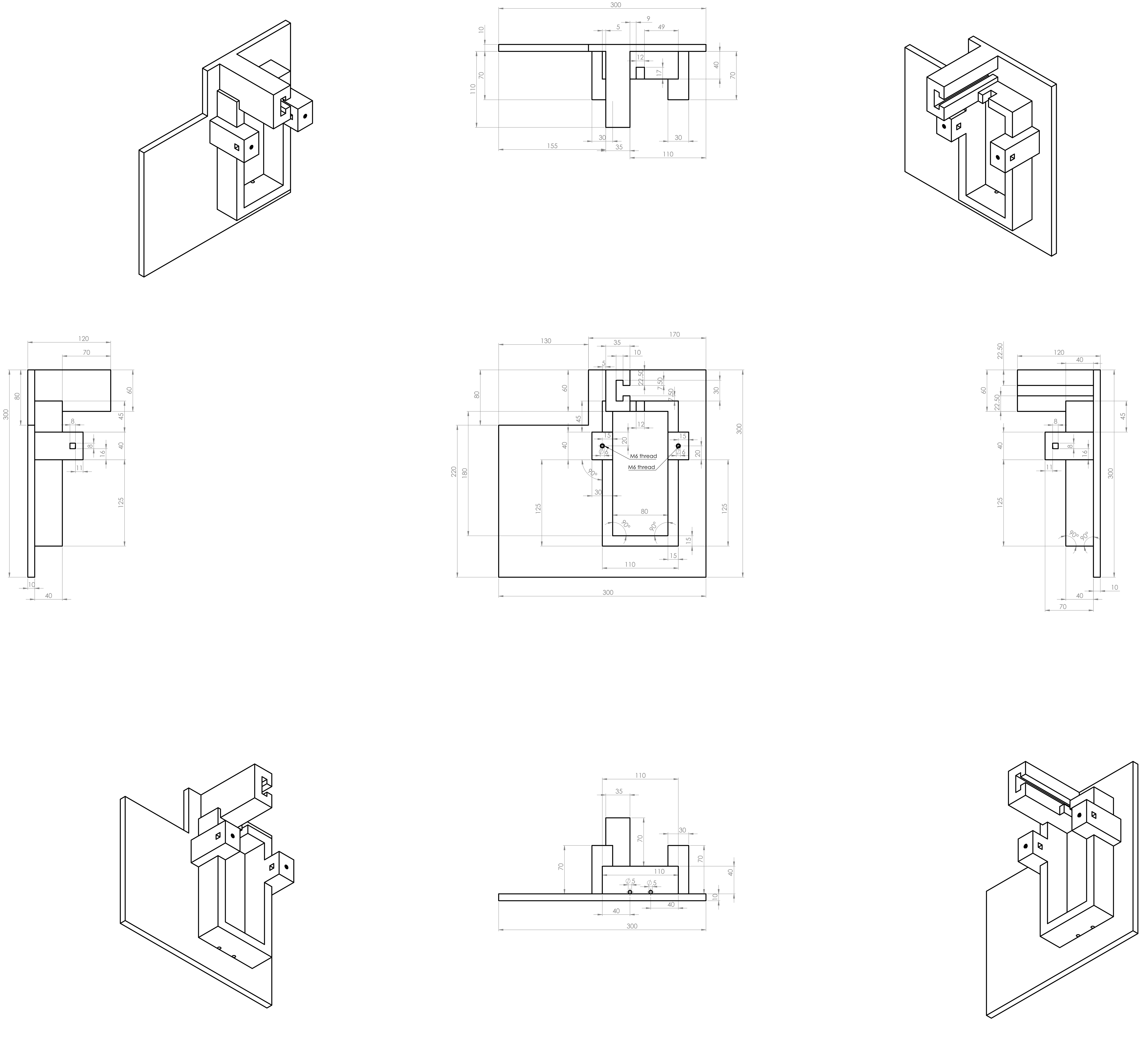

DIMENSIONS IN MILLIMETERS | RECOMMENDED MATERIAL: PMMA | REVISION

	NOM	SIGNATURE	DATE
AUTEUR			
VERIF.			
APPR.			
FAB.			
QUAL.			

TITRE: Recording chamber 2D plan

No. DE PLAN | A0

SCALE: 1:1 | FEUILLE 1 SUR 1

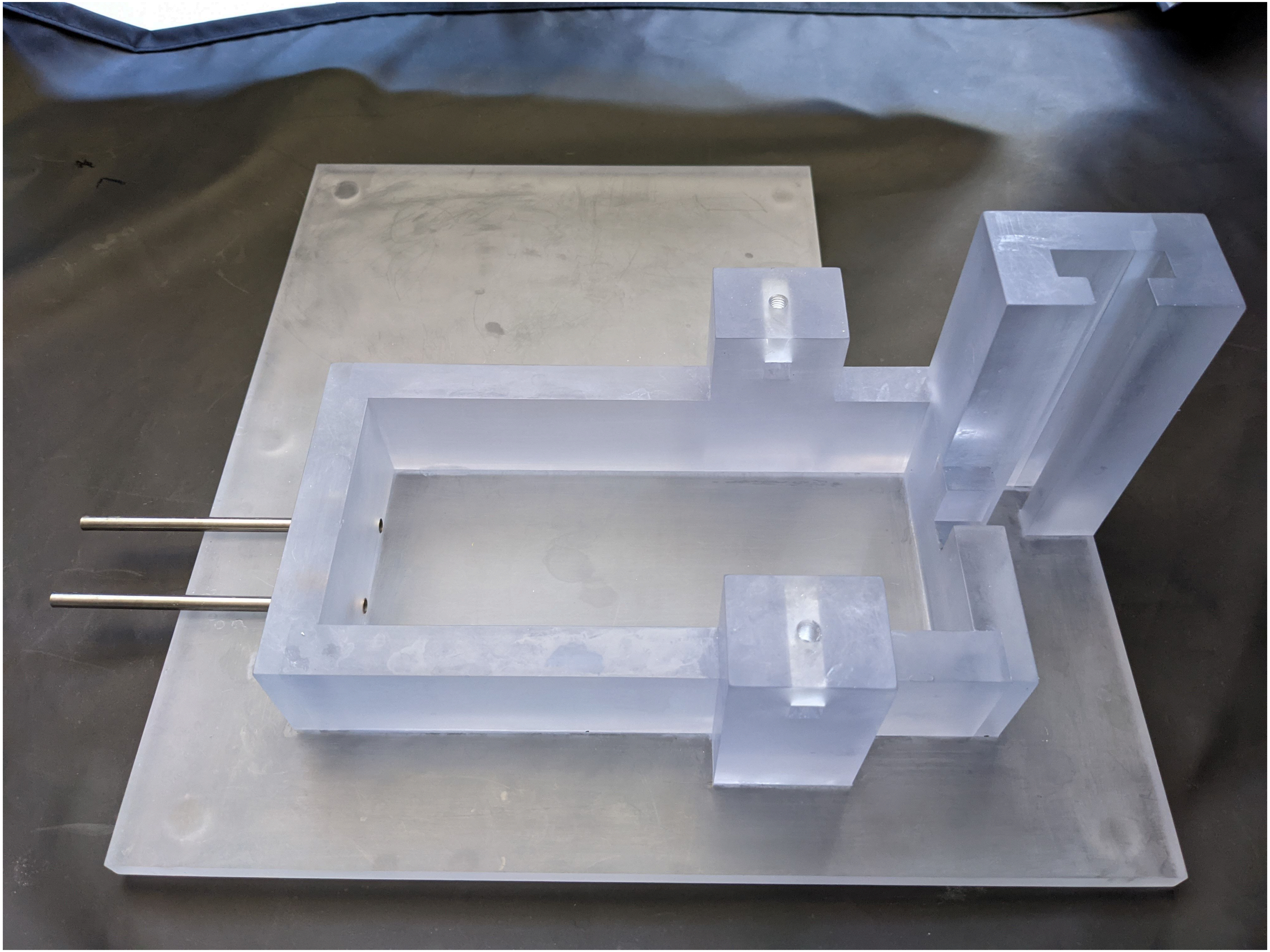

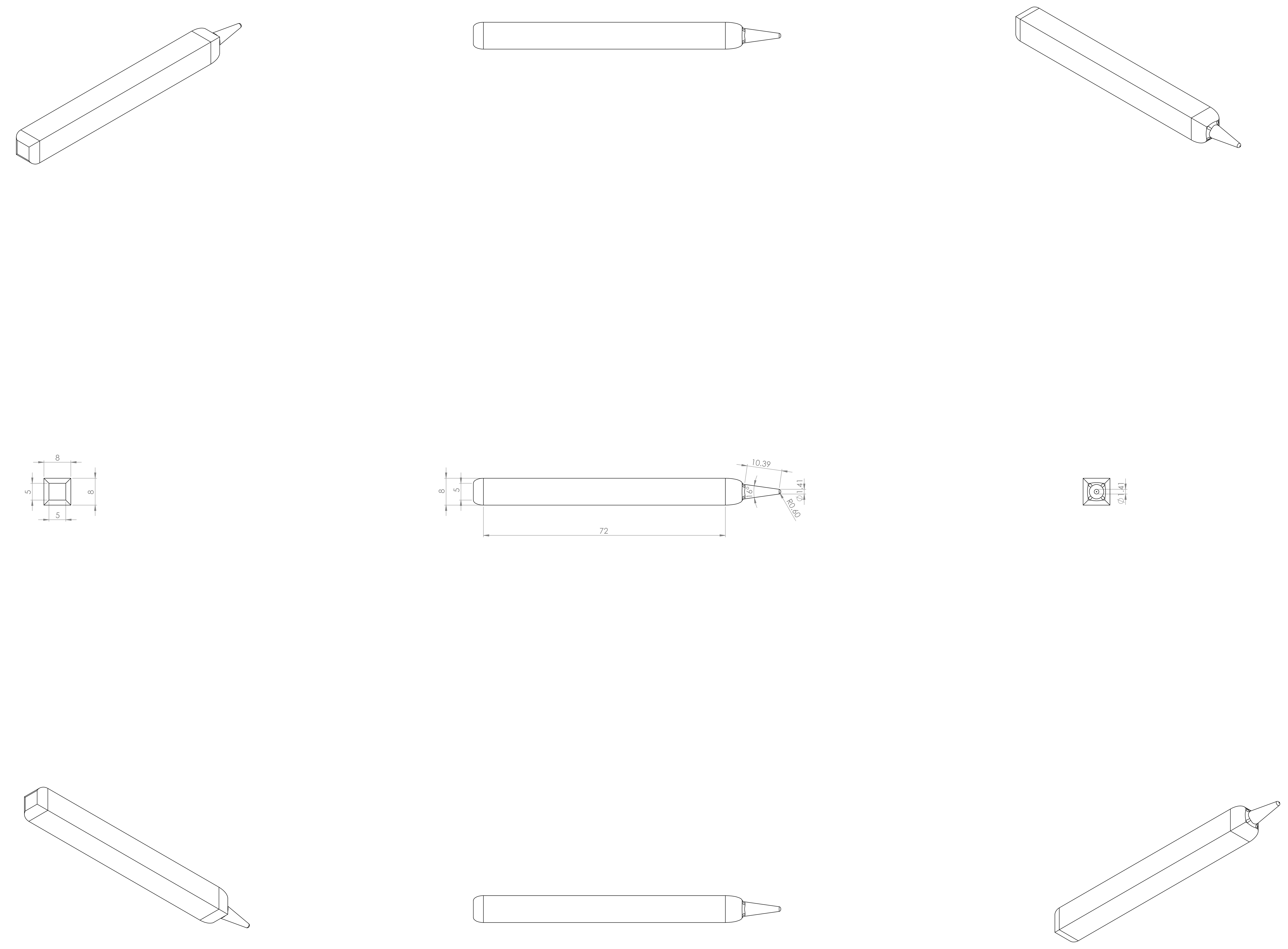

DIMENSIONS ARE IN MILLIMETRES	MATERIAL RECOMMENDED: PMMA		
	NOM	SIGNATURE	DATE
AUTEUR			
VERIF.			
APPR.			
FAB.			
QUAL.			

TITRE:

Ear bar 2D plan

No. DE PLAN

A0

SCALE: 2:1

FEUILLE 1 SUR 1

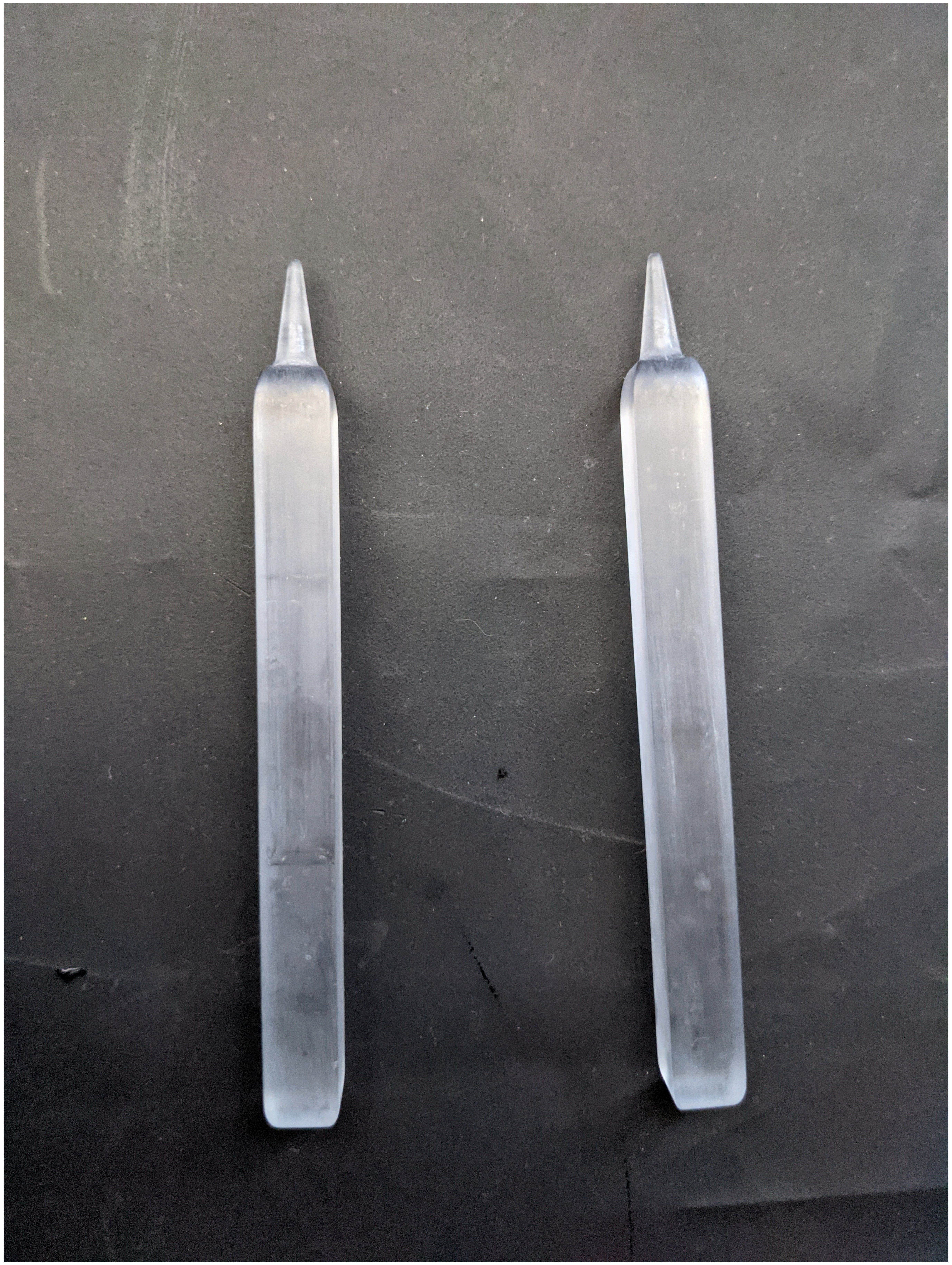

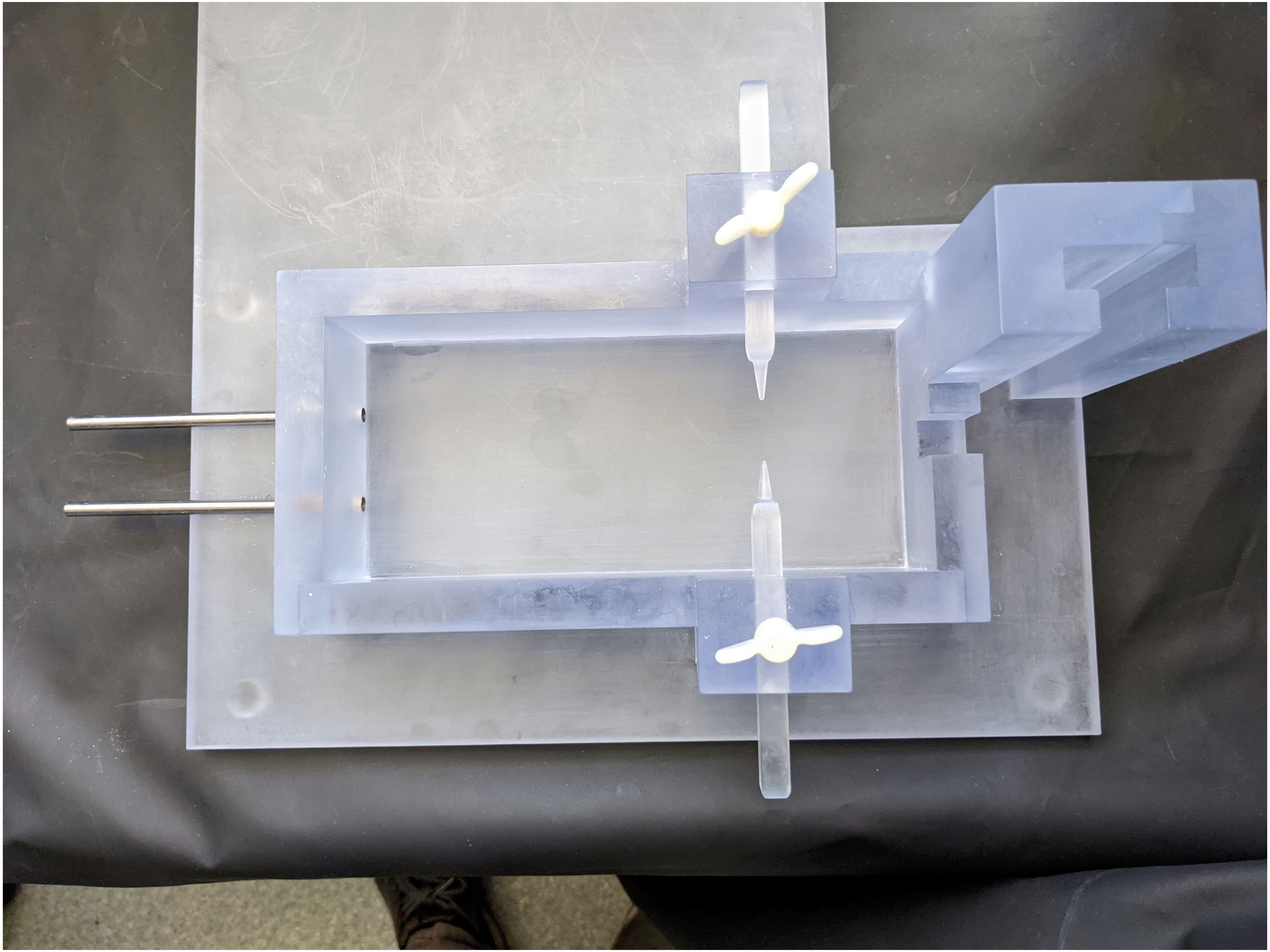

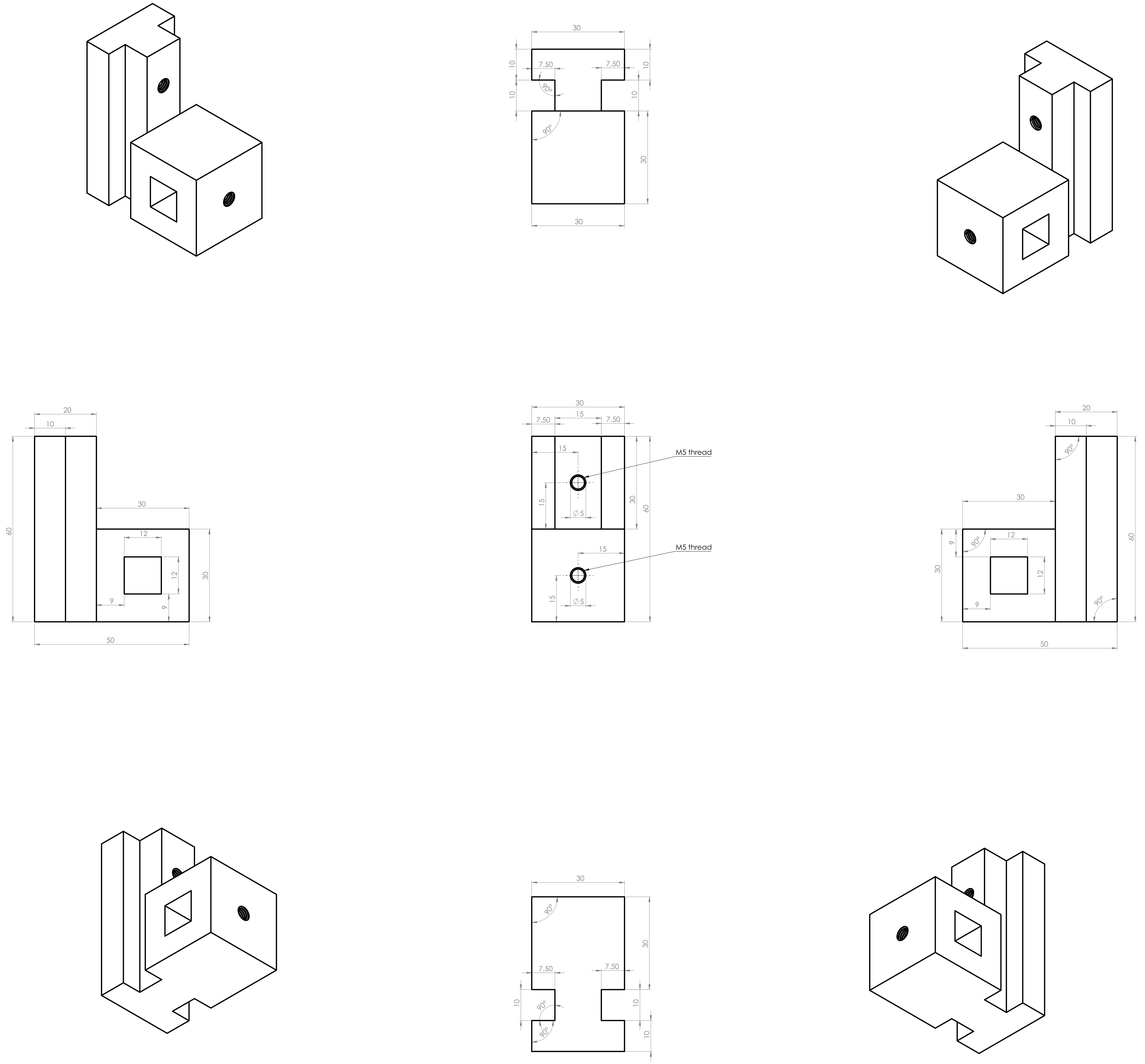

DIMENSIONS IN MILLIMETERS	RECOMMENDED MATERIAL: PMMA		REVISION
	NOM	SIGNATURE	DATE
AUTEUR			
VERIF.			
APPR.			
FAB.			
QUAL.			

TITRE: Mouth bar to recording chamber connecting piece 2D plan

No. DE PLAN

A0

SCALE: 2:1

FEUILLE 1 SUR 1

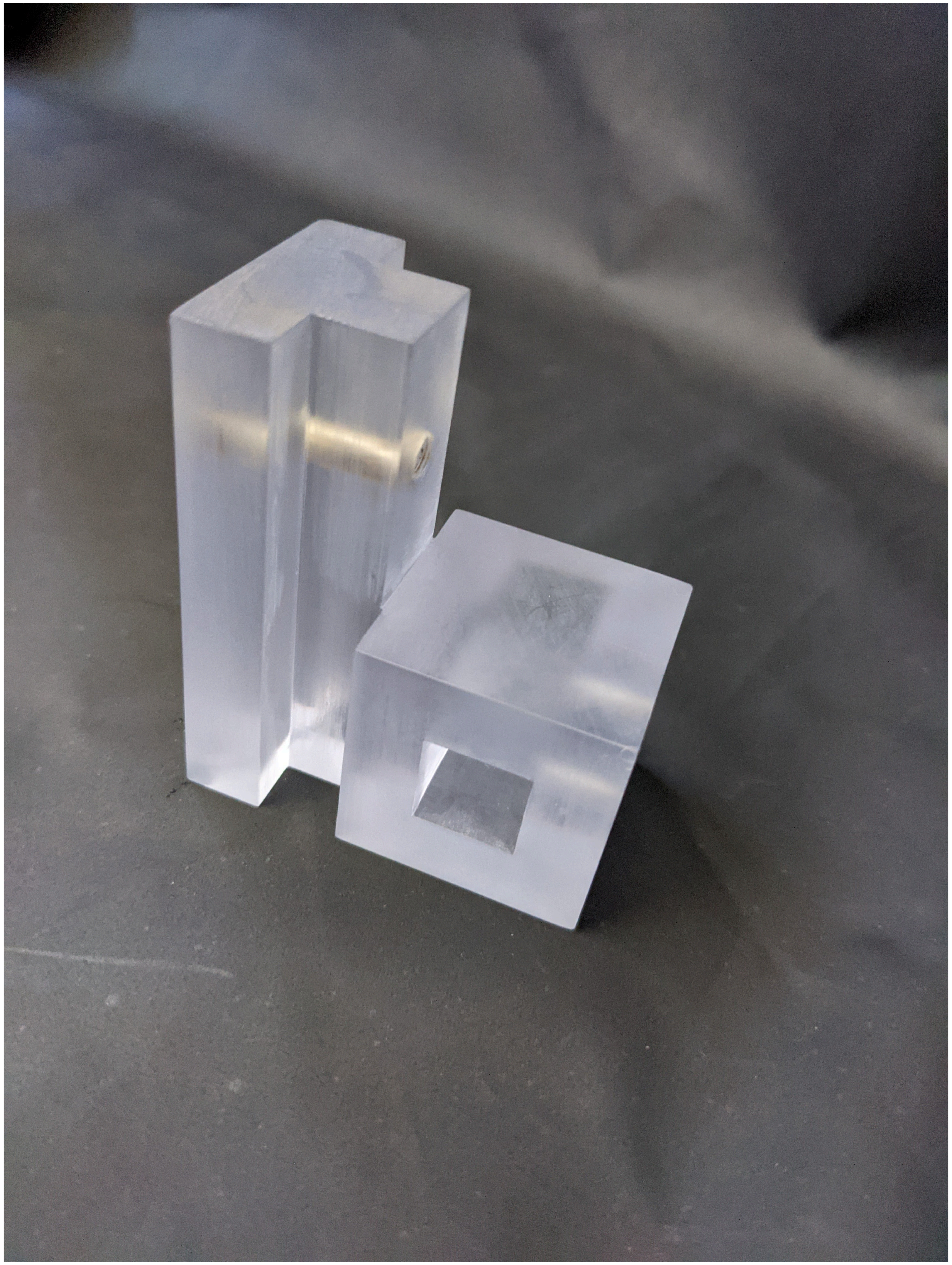

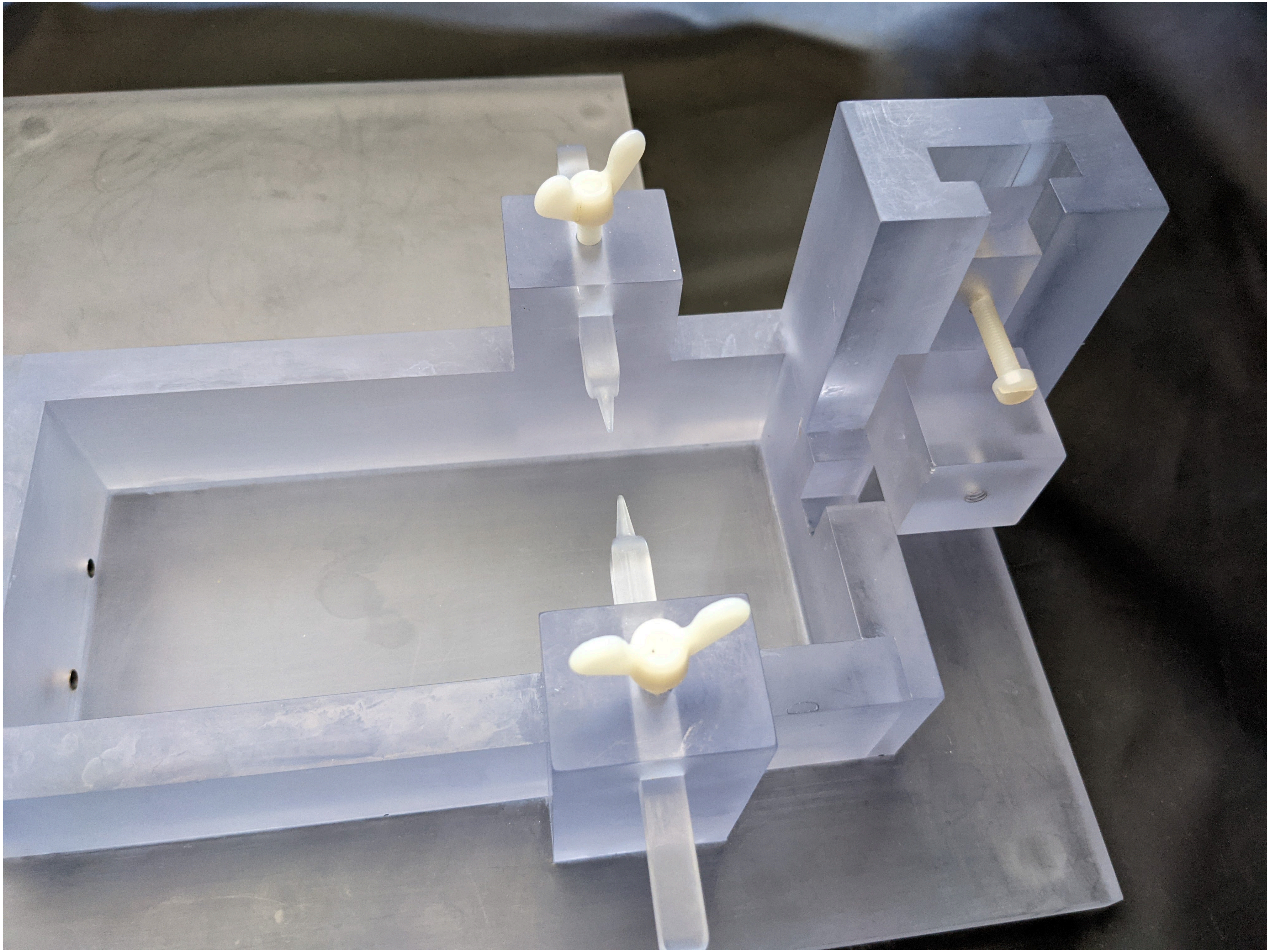

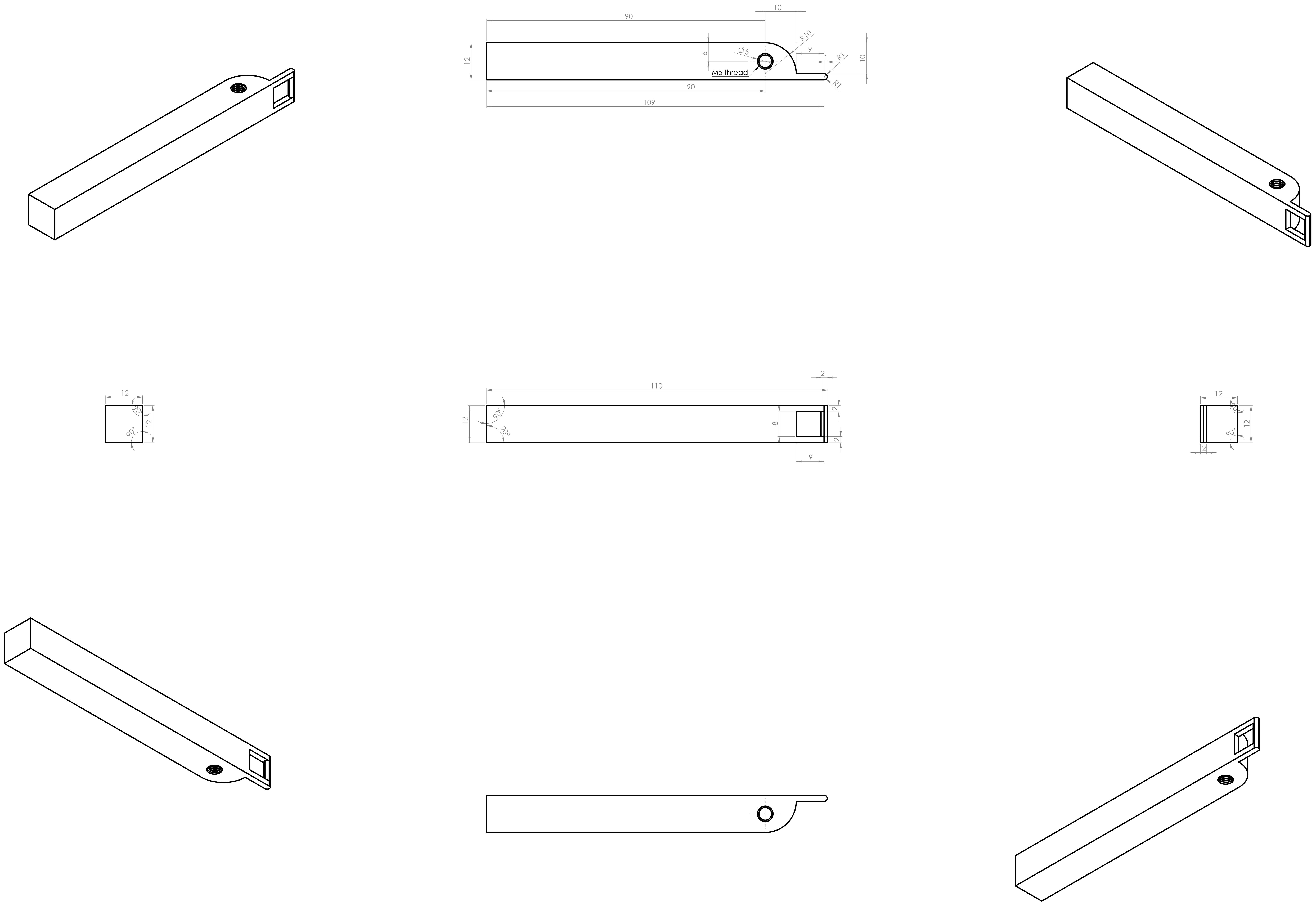

DIMENSIONS IN MILLIMETERS	RECOMMENDED MATERIAL: PMMA		REVISION

	NOM	SIGNATURE	DATE
AUTEUR			
VERIF.			
APPR.			
FAB.			
QUAL.			

TITRE: Mouth bar for recording chamber part 1 2D plan

No. DE PLAN

A0

SCALE: 5:1

FEUILLE 1 SUR 1

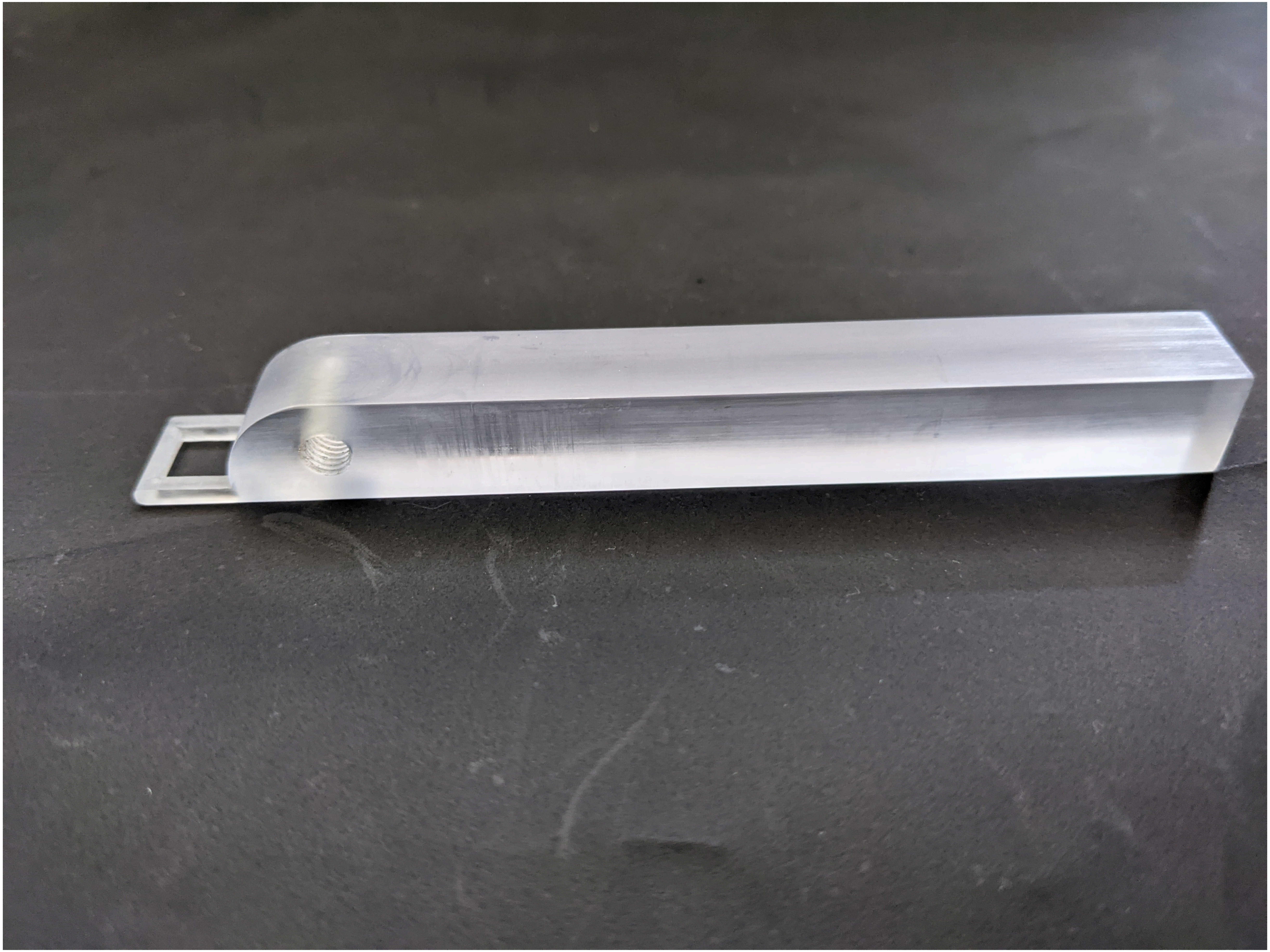

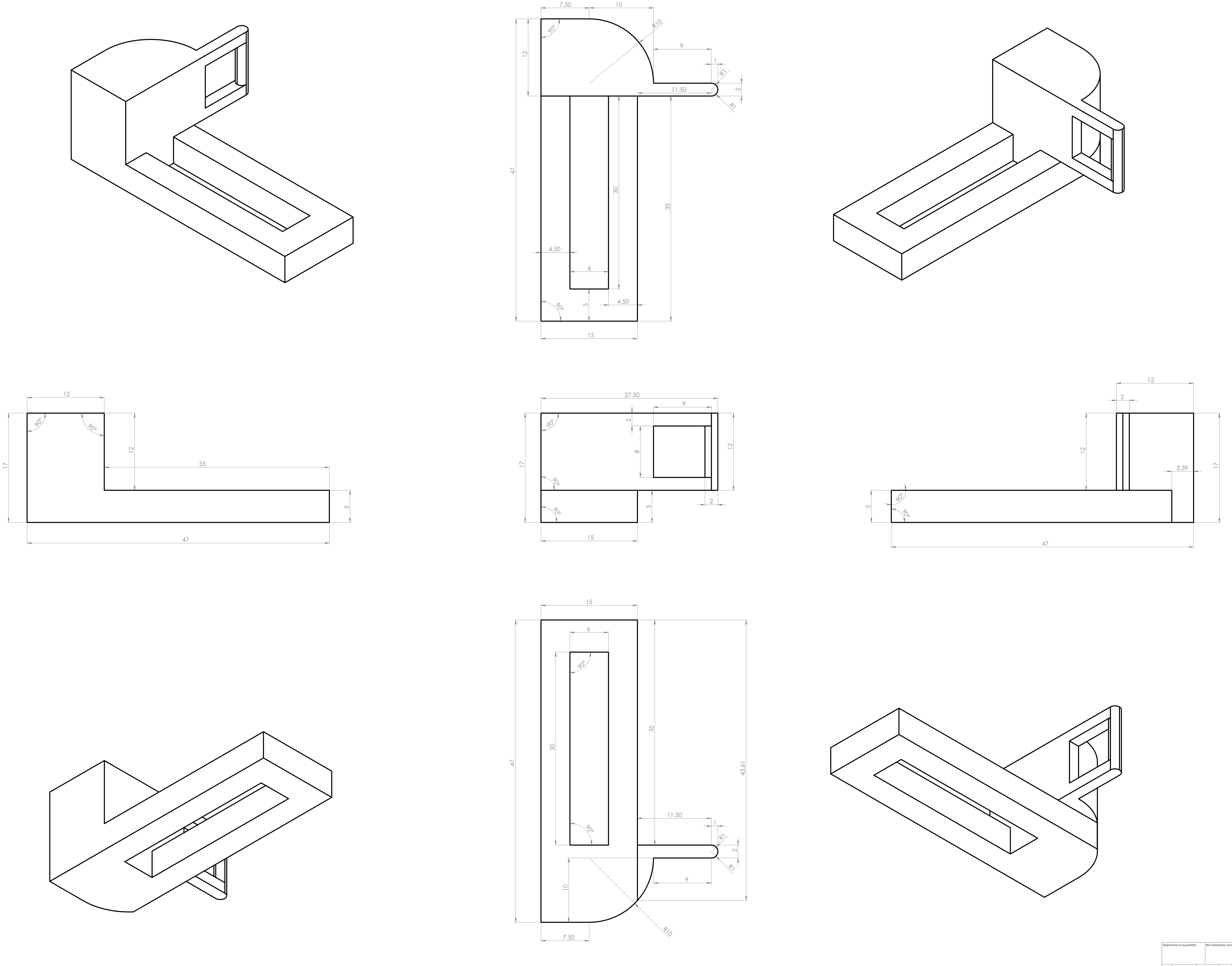

DIMENSIONS IN MILLIMETERS	RECOMMENDED MATERIAL: PMMA		REVISION
	NOM	SIGNATURE	DATE
AUTEUR			
VERIF.			
APPR.			
FAB.			
QUAL.			

TITRE: Mouth bar for recording chamber part 2 2D plan

No. DE PLAN | A0

SCALE: 5:1 | FEUILLE 1 SUR 1

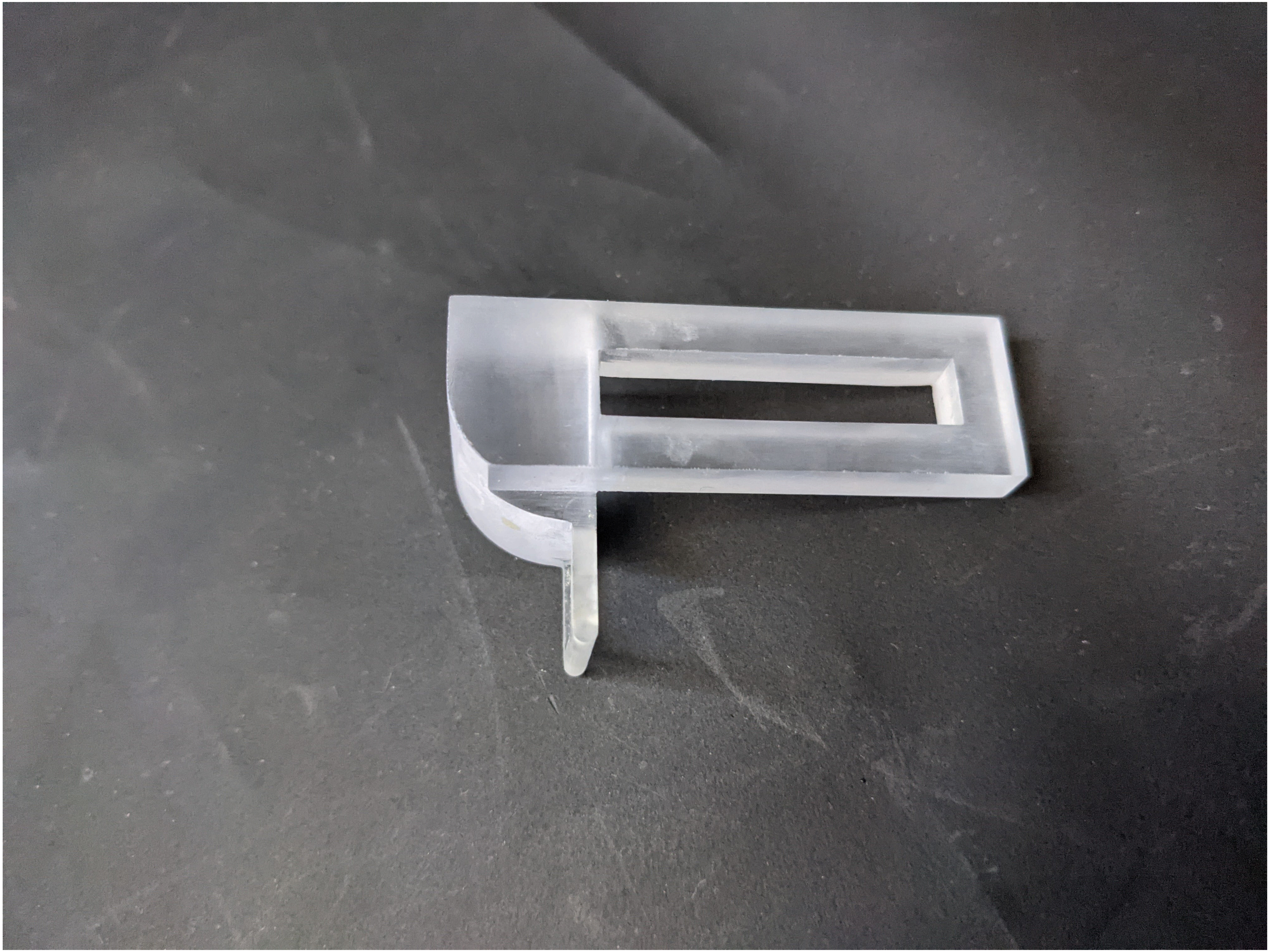

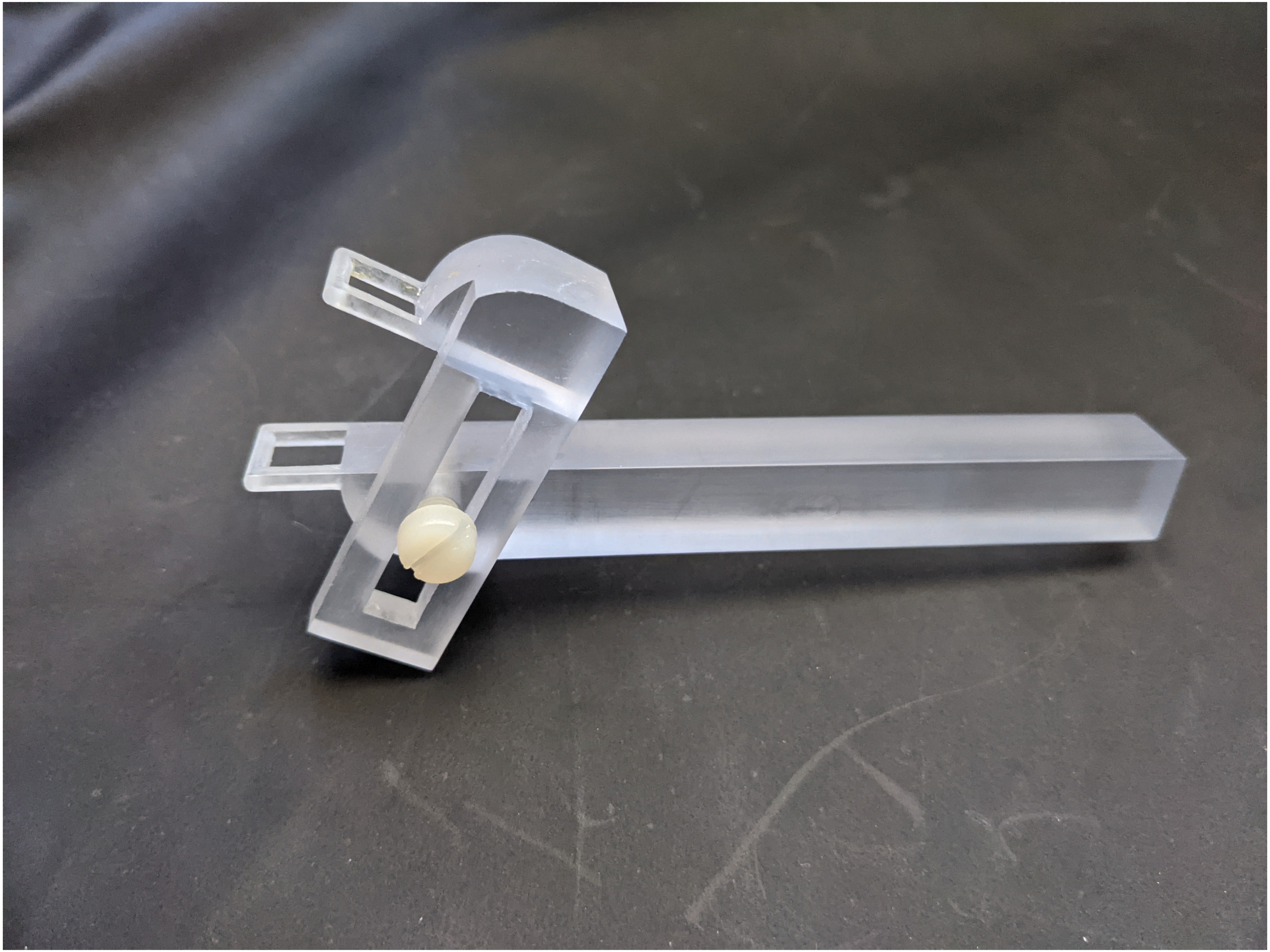

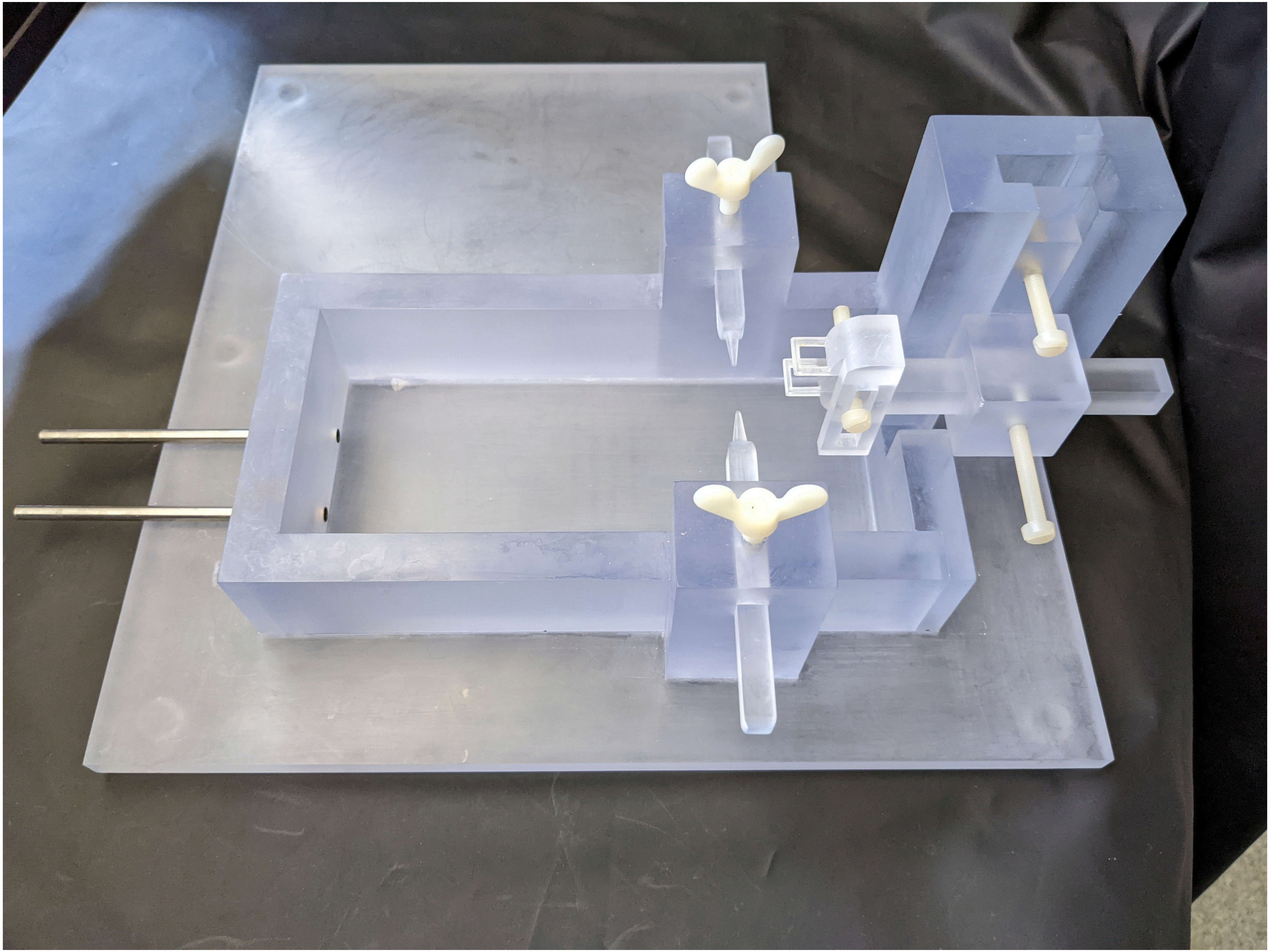

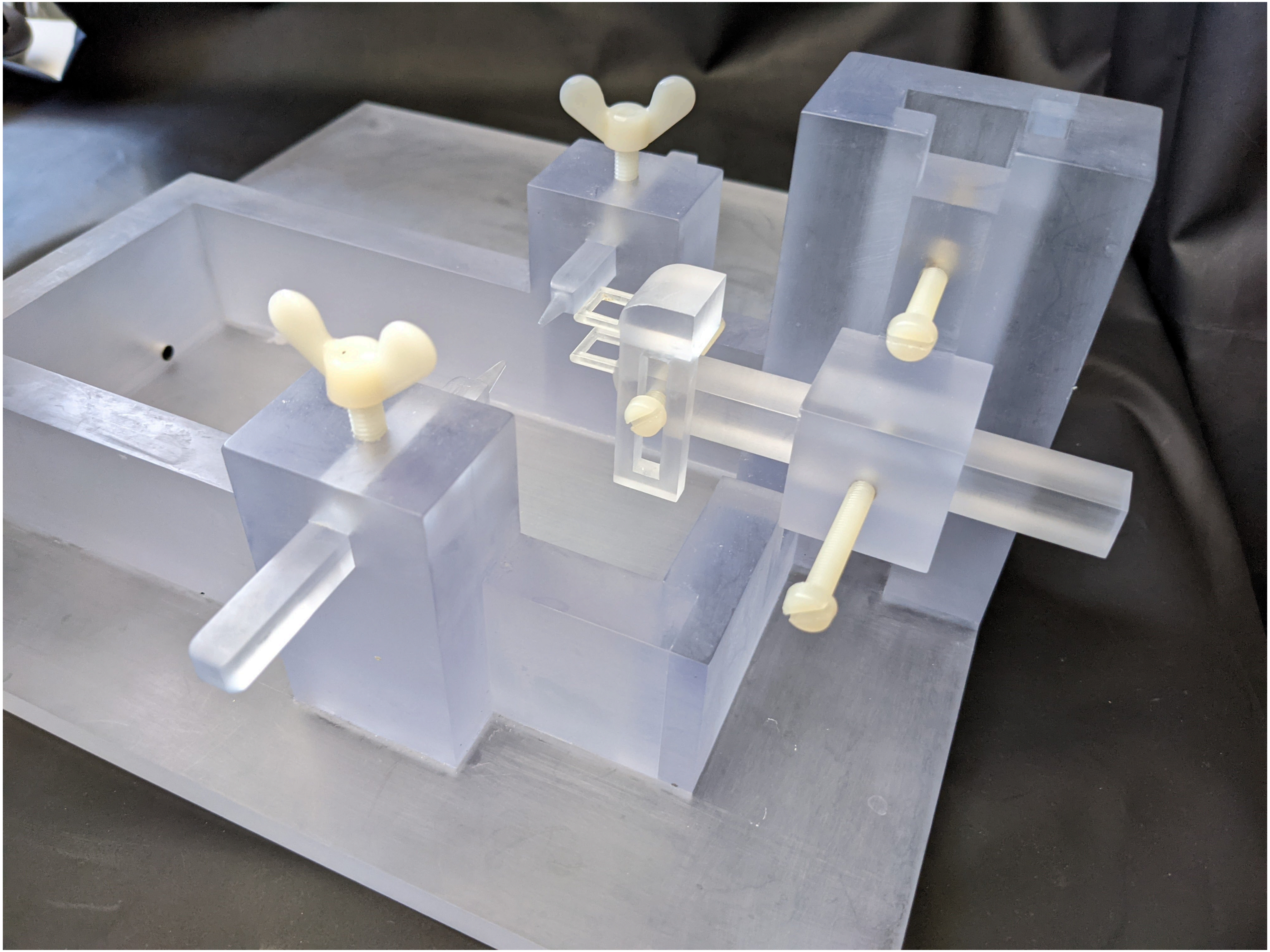

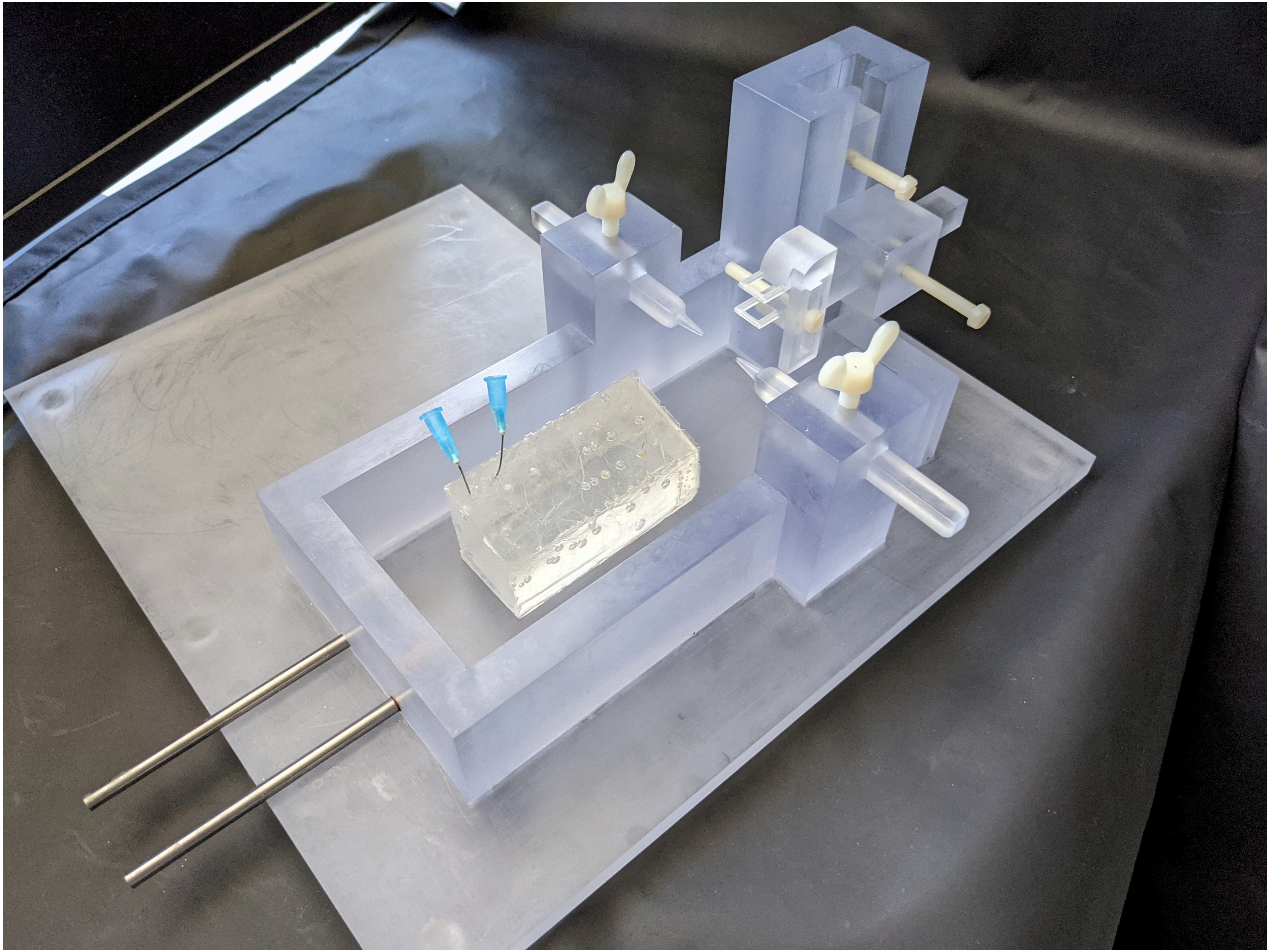

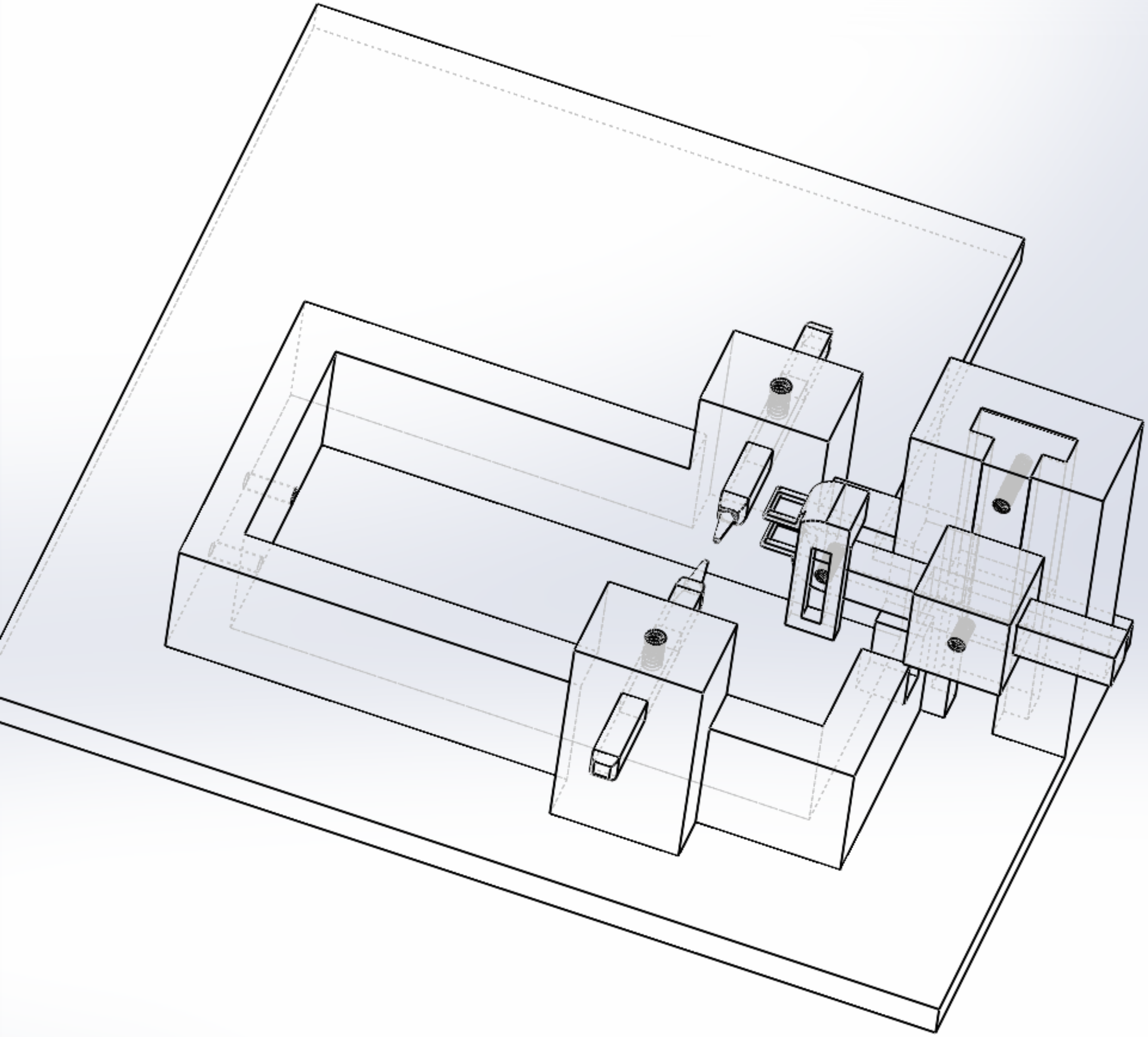

Supplement: Supplementary file 2 — The WHBP set‐up [file TJP-600-2049-s001.pdf]
